# Programming and Dynamic Control of the Circular Polarization of Luminescence from an Achiral Fluorescent Dye in a Liquid Crystal Host by Molecular Motors

**DOI:** 10.1002/anie.202206310

**Published:** 2022-09-05

**Authors:** Jiaxin Hou, Ryojun Toyoda, Stefan C. J. Meskers, Ben L. Feringa

**Affiliations:** ^1^ Stratingh Institute for Chemistry University of Groningen Nijenborgh 4 9747 AG Groningen The Netherlands; ^2^ SCNU-UG International Joint Laboratory of Molecular Science and Displays National Center for International Research on Green Optoelectronics South China Normal University Guangzhou 510006 China; ^3^ Department of Chemistry Graduate School of Science Tohoku University 6-3 Aramaki-Aza-Aoba Aobaku Sendai 980-8578 Japan; ^4^ Molecular Materials and Nanosystems Eindhoven University of Technology 5600 MB Eindhoven The Netherlands

**Keywords:** Liquid Crystal, Achiral Fluorescent Dye, Circular Polarization of Luminescence, Molecular Motors

## Abstract

Circular polarized light is utilized in communication and display technologies and a major challenge is to develop systems that can be switched between left and right circular polarized luminescence with high degrees of polarization and enable multiple addressable stable states. Luminescent dyes in Liquid Crystal (LC) cholesteric phases are attractive systems to generate, amplify and modulate circularly polarized luminescence (CPL). In the present study, we employ light‐driven molecular motors as photo‐controlled chiral dopants in LCs to switch the handedness of the LC and the circular polarization of luminescence from an achiral dye embedded in the mesogenic material. Tuning of the color of the CPL and the retention time of the photoprogrammed helicity is demonstrated making use of a variety of motors and dyes. The flexibility offered by the design based on inherently chiral unidirectional rotary motors provides full control over CPL non‐invasively by light, opening possibilities for pixilated displays with externally addressable polarization.

## Graphical abstract

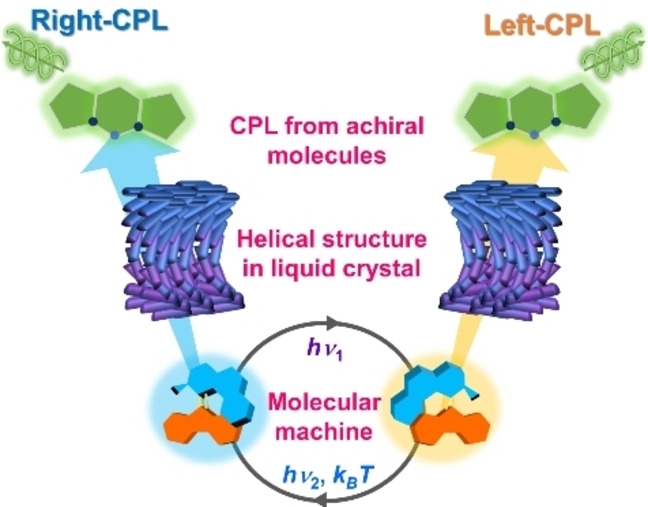

## Introduction

Circular polarization of light carries information and is used in e.g. the animal kingdom,^[1]^ satellite communication^[2]^ and stereographic displays.^[3]^ Circularly polarized light can be generated through luminescence from chiral light emitting materials. The circular polarization of luminescence (CPL) can be quantified by the degree of circular polarization (*g*_lum_) defined as two times the relative difference in intensity of left and right circularly polarized luminescence.^[4]^ Small chiral molecules with allowed optical transitions usually have low |*g*_lum_| values in the order of 10^−5^–10^−3^, which hampers practical application as chiroptical materials.^[5]^ While high *g*_lum_ values have been achieved through design of the molecular structure of organic[^6^, ^7^, ^8^] and inorganic[^9^, ^10^, ^11^] luminophores, it has also become apparent that engineering of the supramolecular helical organization of molecular assemblies allows for high degrees of circular polarization in emission.[^12^, ^13^, ^14^] Particularly challenging is the dynamic control of CPL emission in a fully reversible manner being particularly important toward optoelectronic devices and external addressable materials for display. Attractive opportunities arise from nominally achiral dye molecules in liquid crystal (LC) phases with chiral‐nematic or cholesteric order which can result in highly circularly polarized luminescence from these dyes.[^15^, ^16^, ^17^, ^18^] Here the helicity and helical pitch of the cholesteric phase can be controlled by mixing a chiral dopant into an achiral liquid crystal with local nematic order. The advantage of this strategy is that the helical structure of the cholesteric arrangement and the resulting CPL of the dye can now be manipulated by employing a stimuli‐responsive chiral dopant or LC monomer. It has been shown that control of CPL from LC cholesteric samples can be achieved using heat,^[19]^ electric field,[^20^, ^21^, ^22^] and light[^23^, ^24^] as external stimulus. Control of self‐organized helical superstructures with photoirradiation is fascinating because of its spatiotemporal resolution, cleanliness and relatively low invasiveness to devices.^[25]^ Recently, Li et al. have successfully prepared a photoswitchable luminescent chiral dopant by combining multiple binaphthyl groups with a diarylethene switch core and demonstrated wavelength‐directing CPL.^[26]^ However, current approaches to modulation of CPL helicity lack versatility, easy variation of the emission color, and robustness to maintain a high magnitude of polarization. The persistence time of the induced changes remains challenging. Furthermore, multiple addressable states at distinct wavelengths are highly warranted. For practical CPL applications, such as 3D stereographic displays, an ideal chiroptical switch should meet the following criteria; (i) It should possess multiple states which are thermally stable and can be switched by irradiating at different wavelengths. (ii) The switch should induce inversion of the helicity of the liquid crystal when activated, so that the sign of the CPL changes but the absolute magnitude of the degree of polarization remains large. (iii) Switching should be robust and repeatable. (iv) No interference of switching and excitation of the luminescent dye should occur. (v) The chiral switch should not emit luminescence.

Our group has developed light‐driven molecular motors and chiroptical switches that have multiple chiral states that can be selectively interconverted upon irradiation and which can be addressed in a sequence specific manner.[^27^, ^28^, ^29^, ^30^] A unique feature of these molecular machines is that their various isomeric states have opposite helicities. These photoresponsive chiral molecules featuring intrinsic helicity and combined with controlled change in helicity are particularly attractive as chiral LC dopants due to large helical twisting power (HTP) values and change in (i.e. modulation of) HTP values. When used as switchable chiral dopants in LCs, they can readily switch the handedness of the LC phase between left and right upon irradiaton.[^31^, ^32^, ^33^, ^34^] The use of molecular motor as chiral LC dopant allowed for control and amplification of chirality leading to reorganization of LC films^[33]^ and rotational motion of microscopic objects.^[31]^ A large variety of molecular motors have been developed, which allows us to realize optimal control over the cholesteric helical structure and switching of the LC organization (e.g. handedness).

**Figure 1:**
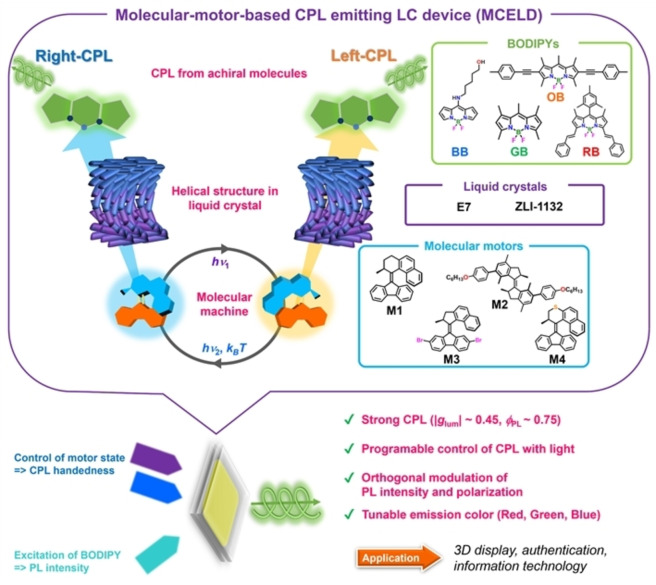
MCELDs (Molecular‐machine‐based CPL‐emitting LC devices) are prepared by mixing molecular motors and BODIPYs in liquid crystal mixtures. This system shows robust CPL switching, orthogonal excitation on molecular motors and fluorescent dyes, tunable CPL color and programmable CPL control.

Based on the favorable chiral and responsive properties, we designed a new LC device emitting CPL; the principle is shown in Figure 1. Here a molecular motor is embedded into the LC as a switchable chiral dopant, in combination with an achiral fluorescent dye. We used BODIPY dyes as luminescent compounds because they have a planar structure, efficient visible light absorption derived from ^1^π‐π* transitions, and bright emission with high fluorescence quantum yields.[^35^, ^36^, ^37^] In addition, their absorption and emission colors can be tuned by chemical modification of their structures. In our mesoscopic material containing molecular motors, LC and dye molecules, the helicity of the particular state of the molecular machine was successfully transferred to the liquid crystalline host, thereby inducing CPL of the dye with large |*g*_lum_| (up to 0.45) and switching between left‐ and right‐CPL. Furthermore, tunability of CPL switching dynamics and CPL emission color was demonstrated by employing different combinations of molecular machines and dyes (Figure 1).

## Results and Discussion

**Figure 2:**
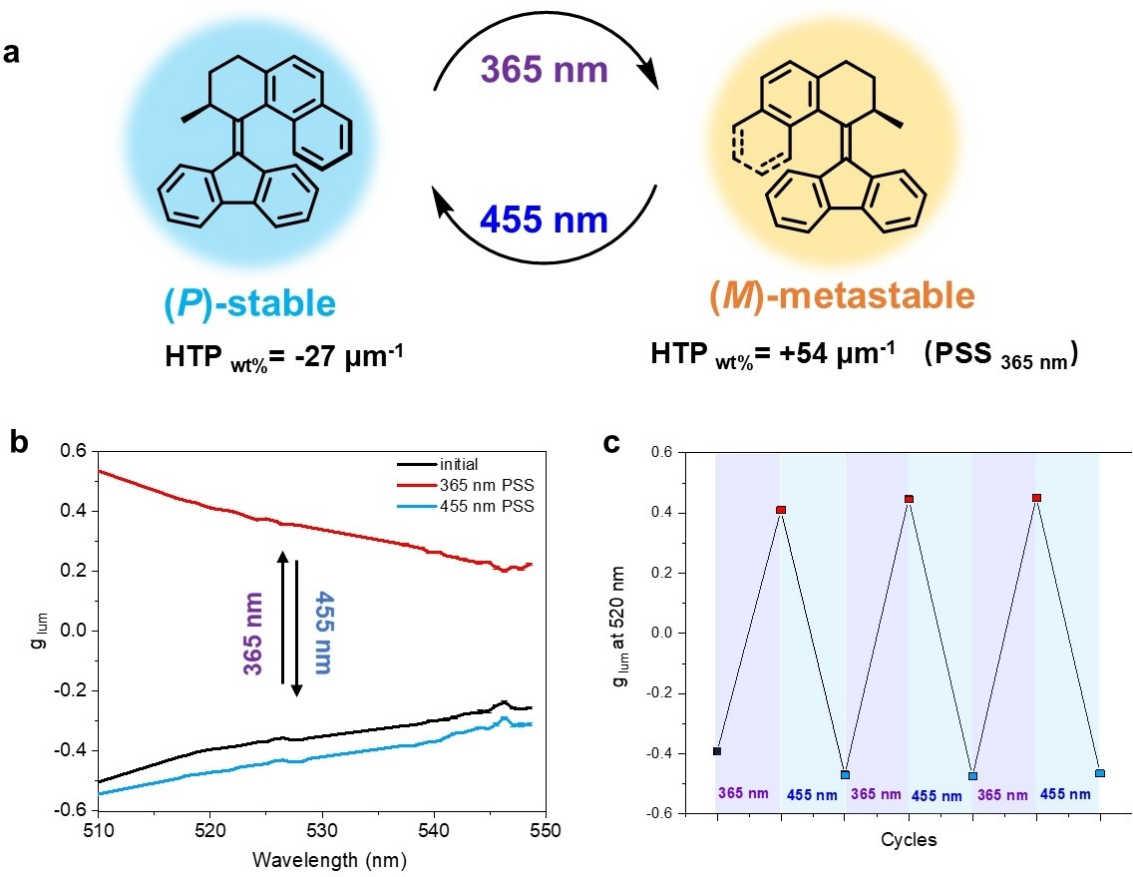
a) Photoisomerization of **M1** and change in HTP values. b) CPL spectral change of **MCELD‐1** at the initial state (black) and after irradiation with 365 nm light (red) and 455 nm light (blue). The device was excited at 470 nm. c) Reversible photoswitching of *g*_lum_ at 520 nm using 365 nm and 455 nm for switching.

**Figure 3:**
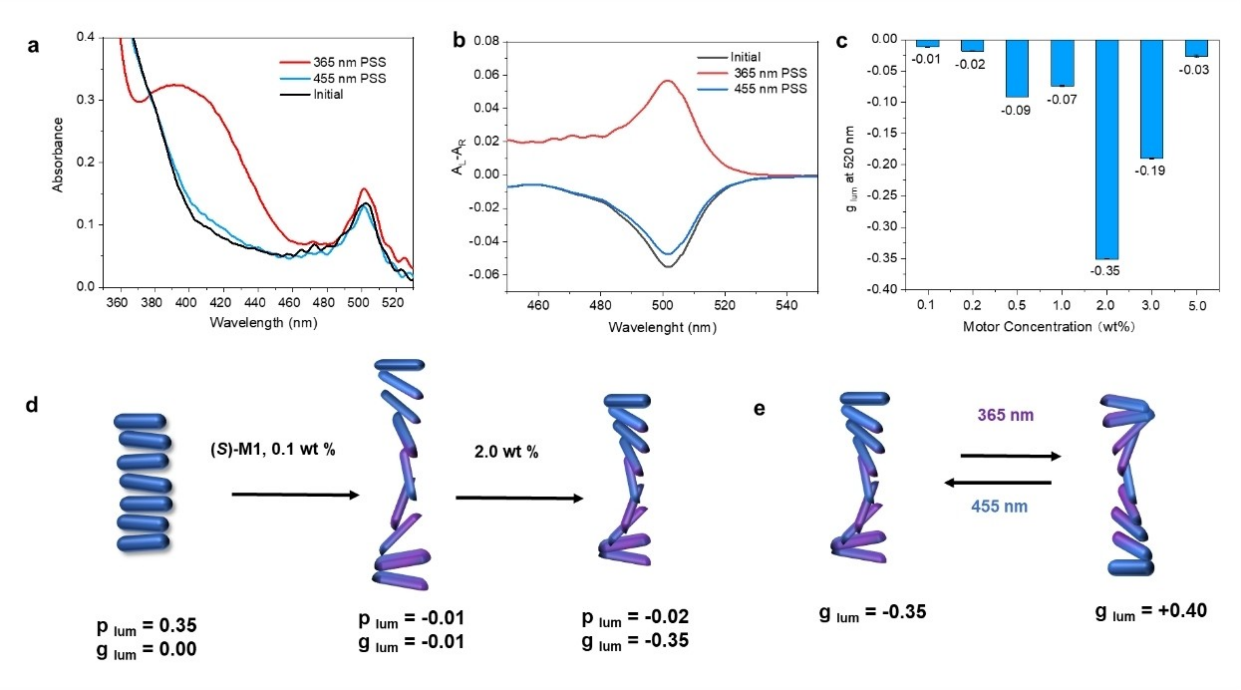
a) UV/Vis absorption spectral change of 1 wt% **M1** in liquid crystal before (black) and after irradiation with 365 nm (red) and 455 nm (blue) LED lamps. b) CD spectral change of 1 wt% **M1** in liquid crystal before (black) and after irradiation with 365 nm (red) and 455 nm (blue) LED lamps. c) **M1** concentration dependence of *g*_lum_ at 520 nm. d) Illustration of LC structures at different **M1** concentrations and e) Helicity and *g*_lum_ change upon light irradiation at 365 nm and 455 nm (2 wt% **M1**). The given *g*_lum_ and *p*_lum_ value are measured at 520 nm.

A favorable motor to apply as a chiral dopant is overcrowded alkene **M1** as our earlier studies on its photoswitching behavior in solution showed excellent reversibility and modulation of *P* and *M* helicity (Figure 2a). When irradiated with a 365 nm UV light, **M1** shows photoisomerization from its stable to metastable state, giving a photostationary state (PSS) with a ratio of (*P*)‐stable: (*M*)‐metastable isomers of 9: 91. The (*M*)‐state has a long lifetime, on the order of 10^3^ years at room temperature, yet irradiation with blue light can retrieve the original (*P*)‐helical state in 20 min.[^38^, ^39^] Based on this study, we have also investigated the photoswitching property of (*S*)‐**M1** as a chiral dopant (1 wt%) in liquid crystal phase using a nematic E7 LC mixture. Upon irradiation with UV light at 365 nm, **M1** switches to its metastable state causing a large change in the molecular organization of the LC phase and, most importantly, inversion of its helicity. The helical twisting power (HTP_wt%_) of **M1** changes from −27 μm^−1^ to +54 μm^−1^ upon illumination (Figure S2, Supporting Information Table 2). Illuminating the metastable isomer with 455 nm light results in efficient electronic excitation of **M1** via its allowed UV absorption band, and induces a new photostationary state with the HTP_wt%_ of −16 μm^−1^, a value close to the initial stable state. These robust photoisomerization features of **M1** as a photoswitchable chiral dopant allow for precise and dynamic control of the chiral nematic organization of the LC. To study modulation of CPL from the LC system, enantiopure molecular machine (*S*)‐**M1** (2.0 wt%) was admixed together with green fluorescent BODIPY **GB** (0.04 wt%) into the commercial liquid crystal E7. It should be emphasized that the lowest absorption bands of **M1** and **GB** hardly overlap (Figure 3a), which is key to achieve orthogonal photochemical modulation of dopant helicity and selective excitation of luminescence. This design feature allows for selective excitation of **GB** with 470 nm light without perturbing the molar ratio between the isomers of **M1** governed by UV illumination. The mixture was used in a quartz LC cell with planar alignment layers^[40]^ resulting in a molecular‐motor‐based CPL‐emitting LC device (**MCELD‐1**) (Figure 2). By allowing the cell to cool from 60 °C to room temperature, a cholesteric organization developed in the LC phase, resulting in a remarkably high CPL from the achiral **GB** with *g*_lum_=−0.35 at 520 nm (Figure 2b). To account for this circular polarization, we anticipate that the LC host material induces alignment of the luminophores such that the initially linearly polarized emission from the dye in combination with birefringence of the surrounding host results in highly circularly polarized emitted light.^[15]^ Upon irradiation of the cell with UV light of 365 nm, the sign of the CPL from **GB** was reversed from negative to positive, resulting in a *g*_lum_ of +0.40. Furthermore, the original negative *g*_lum_ could be recovered almost completely by subsequent irradiation of the cell with light of 455 nm. The control and amplification of chirality from the molecular to the mesoscopic length scale ultimately results in large and robust modulation of CPL emission. This switching of CPL can be repeated several times without any noticeable fatigue, illustrating the robustness of the system (Figure 2c). The switching properties of **M1** in the device **MCELD‐1** meet most of the criteria outlined above, i.e., it possesses multiple thermally stable states that can be addressed with different wavelengths, the switch when activated induces inversion of the helicity of the liquid crystal, via a robust and repeatable switching process while the chiral switch itself does not luminesce, making this design highly promising for future applications. When the (*R*)‐enantiomer of **M1** was used for doping the LC instead of (*S*)‐**M1**, the opposite *g*_lum_ values were obtained, showing that the circular polarization ultimately originates from the molecular chirality of the molecular machine used as dopant (Figure S3). In contrast to recent luminescent liquid crystal systems showing photochemically controlled changes in CPL,[^34^, ^41^] our system can be switched back and forth in a fully optically controlled way. Furthermore, the design presented here involves the interplay of two separate molecules with distinct functions to perform the helicity switching and the luminescence modulation of the device in a mesogenic LC material. This approach allows for greater flexibility and control of luminescent properties because the molecular structures for switching and luminescence can now be optimized independently, as we demonstrate further on.

To explore the large CPL modulation and confirm that the observed switching of CPL in this multicomponent system is driven by the photoisomerization of **M1**, we investigate the molecular machine action in the LC phase in detail. In Figure 3 we show the optical absorption spectrum of the complete **MCELD‐1** cell. The broad absorption band centered around 420 nm (*λ*_max_=420 nm for metastable state in hexane^[38]^), which becomes visible after irradiating the cell with 365 nm light for 5 min, is characteristic of the metastable state of **M1**. The absorption band disappears upon subsequent irradiation with 455 nm light. The effectiveness of this UV irradiation sequence to switch the cell can also be illustrated by observing the change in color through a polarizing optical microscope (POM, Figure S4). These observations together with the absence of the 420 nm absorption band in the pristine state provide compelling evidence for the photoisomerization of **M1** in the LC material. The absorption spectrum also shows the contribution of the ^1^π–π* transition of BODIPY **GB** as a separate peak at 500 nm. While the absorption by the dye is largely unaffected by irradiation with UV light, the circular dichroism (CD) in the region of the dye absorption shows a reversible inversion of sign following the isomerization of **M1** (Figure 3b). When **M1** is in the metastable state after 365 nm irradiation, the CD absorption at 500 nm is positive, while after irradiation with 455 nm light or in the pristine state the CD signal is negative, which is testimony of induced CD in the supramolecular system transmitted from motor **M1** via LC E7 to fluorescent dye **GB**. This sign alternation of the CD displayed by the achiral dye upon switching of **M1** is consistent with inversion of the handedness of the LC and the embedding of the aligned dye in the chiral nematic host structure. As a result, the helicity of the molecular motor controls the chiroptical response, specifically the induced CD and CPL of the achiral dye. We note that in these optical experiments, the concentration of **M1** was lowered to 1.0 wt%, to stay within the limits of detection of the CD spectrometer. Measurement of the circular differential transmission using cells containing 2.0 wt% of **M1** also confirmed the sign inversion (Figure S5). The concentration of **M1** has a strong influence on the CPL of the dye in the device (Figure 3c and Figure S6–S11) The motor concentration was varied from 0.1 to 5.0 wt% in a 25 μm‐thick cell. The largest *g*_lum_ value of −0.35 was achieved with a concentration of 2.0 wt%. At low concentrations of **M1** (0.1, 0.2 and 0.5 wt%), almost no CPL was observed but instead the cell shows significant linear polarization of luminescence (LPL). The degree of linear polarization can be quantified using $p_{lum}=2\frac{I_{\left|\right|}-I_{⊥\;}\;}{\;I_{\left|\right|}+I_{⊥\;}\;},$ where $I_{\left|\right|}$ and $I_{⊥}$ represent the intensity of luminescence polarized parallel and perpendicular to the rubbing direction of the alignment layers in the cell. When the chiral dopant **M1** is absent or present in only low concentrations, the LC is likely to order nematically with the mesogens oriented in the direction set by the alignment layers.[^42^, ^43^] The high LPL of the dye emission supports the alignment of the chromophores in the LC host. When the concentration of **M1** reaches 2.0 wt%, LPL becomes vanishingly small and strong CPL emission was achieved, indicative of the formation of the helical superstructure. Polarized optical microscope (POM) observation clearly showed the emergence of the cholesteric phase when the concentration is higher than 1.0 wt%. When increasing the loading of the LC with **M1** to 3.0 and 5.0 wt%, *g*_lum_ decreased relative to the value at 2 wt%. This deterioration of the circular polarization properties of the luminescence at high loading is attributed to aggregation of the motor, as indicated by dark spots in the POM images (Figure S11). Finally in an effort to optimize the circular polarization while suppressing the unwanted linear polarization, the thickness of the cells has been varied. With thin cells (3 μm and 6.5 μm), relatively large LPL was observed while LPL became less noticeable when thicker cells (10, 18 and 25 μm) were used. The loss of linear polarization most likely occurs on a mesoscopic scale, involving birefringence and formation of multiple domains. Gratifyingly, CPL itself did not show a strong dependence on the thickness, indicating that the CPL is mainly generated locally, i.e. within a limited volume of mesoscopic dimension around the dye molecule.

**Figure 4:**
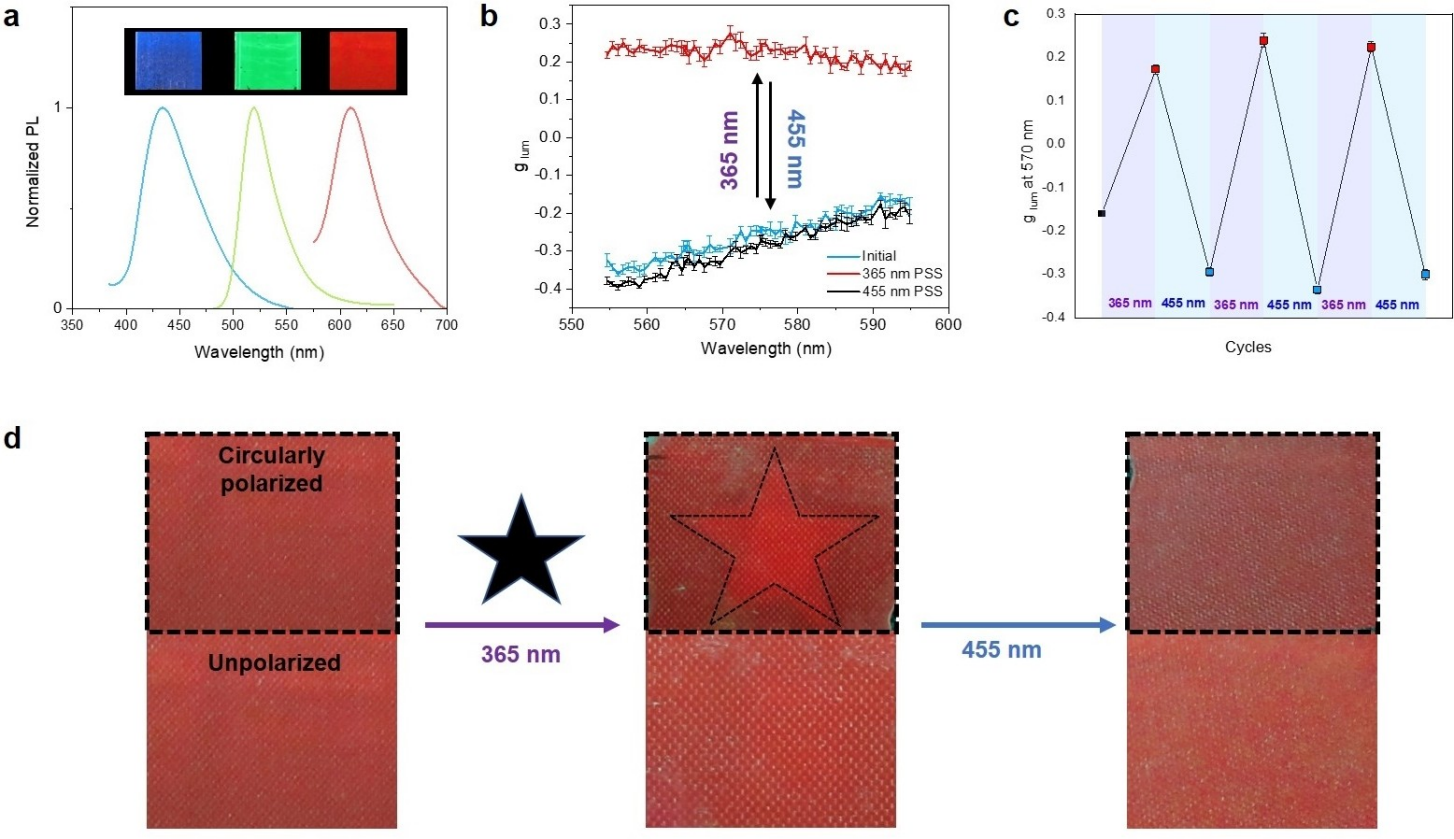
, a) Normalized PL spectra of **MCELD‐1** (green, **GB**), **MCELD‐2** (orange, **OB**) and **MCELD‐4** (blue, **BB**). The devices were excited at 460 nm, 530 nm and 365 nm, respectively. b) Photoswitching of *g*_lum_ of **MCELD‐2** (orange, **OB**) upon irradiation with 365 nm or 455 nm light. c) Reversible CPL switching with 365 nm and 455 nm irradiation. The irradiation cycle was repeated three times. d) Observation of CPL switching with (top) and without circular polarizer (bottom). The sample was excited with 470 nm and observed through an optical longpass filter (*λ*>500 nm).

A distinct feature of our design is the high flexibility in controlling the photoactive layer. The molecular machine and the fluorescent dye can easily be adapted because there is no direct interdependency between machine and dye structure, as they are separate entities in contrast to earlier approaches in the generation of the polarized light. With the broad choice of molecular machines and fluorescent molecules available, tuning of the wavelength (color), intensity and sign inversion of CPL is readily achieved. In addition to the green‐luminescent **GB**, blue‐, orange‐ and red‐emissive BODIPYs (**BB**, **OB**, and **RB**, respectively; Figure 1) were prepared and incorporated in LC devices to demonstrate tunablity of color in CPL switching (Figure 4a). Typically, 0.04 wt% of **OB** and 2.0 wt% of **M1** were added to E7 and the resulting LC material was filled into a 25 μm‐thick planar cell (**MCELD‐2**). Upon 365 nm irradiation, *g*_lum_ at 570 nm changed from −0.30 to +0.25, and reverse CPL switching could be induced with 455 nm light (Figure 4b). Thus, by using dye **OB** with extended π‐system, the color of the circularly polarized emission can be shifted from green to orange, while maintaining a CPL switching behavior similar to **MCELD‐1** (Figure 4c). Remarkably, the contrast in circular polarization upon switching is now so large that it can even be perceived by eye when the luminescent film is viewed through a circular polarizer (CP, Figure 4d and Figure S12). To demonstrate this feature, **MCELD‐2** was irradiated through a star‐shaped photomask with a 365 nm LED lamp for 5 min. In the illuminated area, **M1** is switched to the metastable state. After completing the photopatterning and removing the mask, the part that has been irradiated appears brighter when viewing the luminescent layer through a left circular polarizer and exciting with 470 nm light. With the molecular motor switched to the metastable state, this part of the film emits predominantly left circularly polarized light (*g*_lum_>0). The unirradiated parts still emit largely right polarized light (*g*_lum_<0) and appear darker when viewed through the polarizer. Without the polarizer, the emission from the device appears homogeneous. The star‐shaped pattern can be erased by irradiation with 455 nm light for 15 min. This reversible photopatterning highlights the potential for dynamic and spatially resolved control of CPL.

Red luminescence with emission maximum at 640 nm has been realized by using the dye **RB**. Yet a device with **RB** and **M1** in E7 (**MCELD‐3**) shows a much smaller *g*_lum_ value (−0.04 at 640 nm) when compared to **MCELD‐1** and **MCELD‐2**. Here, the circular polarization emitted by the **RB** dye shows only minimal variation after irradiation with 365 nm UV light (Figure S13). We anticipate that the alignment of the extended **RB** dye in the LC host may differ from that of the other dyes.

Blue luminescence can be provided through dye **BB**. The lowest allowed absorption band of this dye is in the 370 to 450 nm wavelength range. This absorption band overlaps with the absorption of molecular machine **M1**. Therefore, in order to have stable blue emission from a layer with **BB**, a different molecular machine needs to be used with absorption bands below 370 nm. Molecular machine **M2** does not absorb significantly above 370 nm and can be switched with 312 nm and 365 nm irradiation (Figures S14–S17).^[33]^ Device **MCELD‐4** was prepared by using a mixture of **BB** (0.04 wt%) and **M2** (1.0 wt%) in LC mixture ZLI‐1132 into a 25 μm‐thick LC cell. Upon irradiation with 312 nm light for 30 min and then 365 nm light for 10 min, **M2** switches from the stable *cis* state to the unstable *cis*‐conformer and subsequently converts to the stable *trans*‐state. These three different states of the machine give rise to a *g*_lum_ of +0.02, +0.08 and −0.05, respectively for the blue emission from the **BB** dye in **MCELD‐4** at 455 nm.

In summary, BODIPY‐type dyes offer high photoluminescence quantum yields in the LC matrix (up to 75 %, Figure S18) and allow for color tuning of the emission. The circular polarization of the dye emission and its switching ability can be controlled through the structure of the dye and the chiral molecular motors.

A large library of molecular machines that change molecular helicity in response to an external light stimulus is now available from our research program.[^27^, ^28^, ^29^, ^30^] The variation in rates for transitions between various states of these molecules allows us to modify the nature and dynamics of the circular polarization response. This resulted in the discovery of fast response times in the time dependence of the CPL when exerting dynamic control. To further illustrate this tunability, two additional molecular machines **M3** and **M4** were explored. These motors were combined with dye **GB** and the E7 LC to yield devices **MCELD‐5** and **MCELD‐6**, respectively.

**Figure 5:**
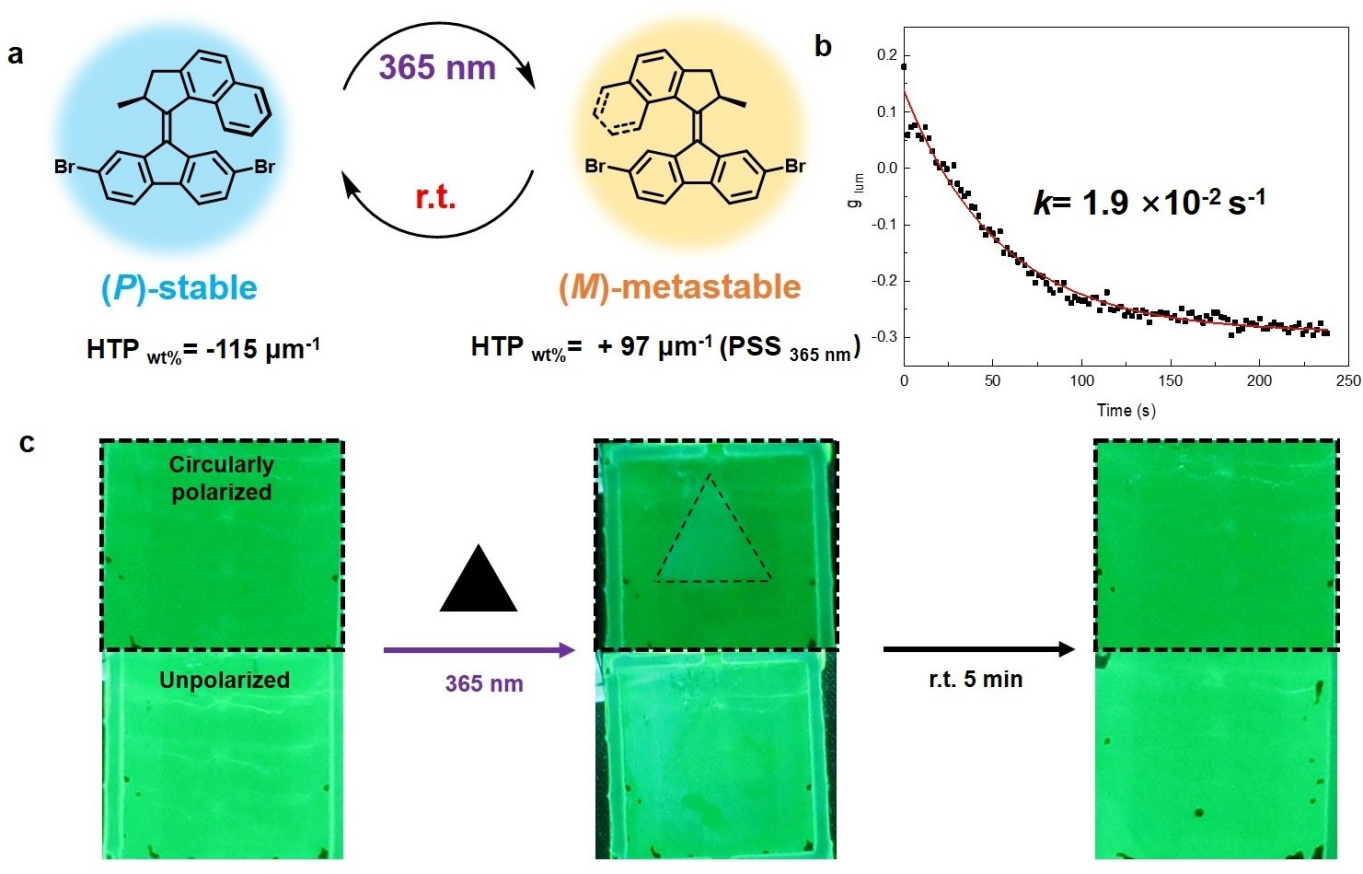
a) Photoisomerization of **M3**. b) Time‐dependence of *g*_lum_ recorded at 522±6 nm directly after termination of prolonged irradiation with 365 nm light at *t=*0. The device was excited at 470 nm. c) Observation of CPL switching with (top) and without (bottom) a circular polarizer. The sample was excited at 470 nm and a longpass filter was used to cut off light with wavelength<500 nm.

**M3** is a molecular motor (Figure 5a) which has a short‐lived, metastable state that can be induced by irradiation with 365 nm light. In solution, this transient state reverts back to the stable conformer with a lifetime of 3.5 min at room temperature. **M3** is also active in the device **MCELD‐5** (Figure S19). Photoisomerization of **M3** in the LC mixture upon 365 nm light irradiation, is evidenced by a shift of the maximum of the absorption band of **M3** from 400 nm to 420 nm, corresponding to the transition from the stable to the metastable state. Subsequently, in the dark, **M3** as a dopant in the LC matrix went back to its initial stable state within 5 min, via a thermal inversion as shown by the recovery of the initial absorption band. Transient changes in the sign of the CD in the absorption region of **GB** during this cycle indicate that the motor can induce a temporary inversion in the handedness of the cholesteric arrangement (Figure S19). The luminescence of **GB** in the stable state of **MCELD‐5** is predominantly right circularly polarized with *g*_lum_=−0.30. Irradiation of **MCELD‐5** with UV light of 365 nm wavelength for 5 min resulted initially in an inversion of the sign of the CPL for the green emission from **GB** near 520 nm followed by the gradual recovery of *g*_lum_ from +0.20 to −0.30 in a period of 250 s (Figure 5b). The kinetics of recovery follows a single exponential curve with a rate constant of 1.9×10^−2^ s^−1^, corresponding to a lifetime of 53 s. Here, the transient change in circular polarization of luminescence can also be observed directly by eye when viewing the emission through a circular polarizer (CP, Figure 5c). The device with **M3** and **GB** was first irradiated with 365 nm through a photomask with a triangular‐shaped hole and brought to the PSS. After terminating the UV irradiation, removing the mask, exciting with 470 nm and viewing the layer through the CP, a triangle with increased brightness compared to the surrounding film area directly revealed the difference in CPL between the irradiated and nonirradiated areas. Over time, the pattern gradually faded within 5 min and the homogeneous green luminescence was recovered. This example together with the results shown in Figure 4 illustrate the high level of control over the photoinduced change in polarization properties of the luminescence that can be achieved through molecular engineering of the chiral motor and the combination of photoresponsive dopant and luminescent dye.

Finally, we found that molecular machine **M4** shows quasi‐irreversible photoisomerization in the LC matrix (Figures S20 and S21). **M4** in its thermodynamically most stable state induces a negative *g*_lum_ value for the emission of **GB** in the LC mixture (−0.08 at 510 nm). Irradiation of the device with 365 nm light resulted in a large change of *g*_lum_ to positive values (+0.45). Yet, neither a spontaneous nor a thermal or photoinduced reversion of *g*_lum_ to its original negative values was observed within the time frame of the experiment. This apparent impossibility of recovering the initial state is most likely related to the large differences in helical twisting power of the various isomers of this particular machine in the E7 liquid crystal host. While for **M4** in its thermodynamically most favoured state we find a relatively small negative HTP_wt%_ of −8 μm^−1^, the HTP_wt%_ for the PSSs of the machine under illumination with 365 and 455 nm are positive and much higher, +54 and +17 μm^−1^, respectively. Thus, the presence of a relatively small fraction of **M4** in a metastable state may already induce helicity in the LC opposite to that induced by the pure, thermodynamically most stable state. These quasi‐irreversible, photoinduced changes in devices with **M4** may find application in authentication.^[44]^

## Conclusion

In summary, we have constructed customizable light‐emitting devices whose circular polarization can be controlled dynamically through the conformational state of embedded molecular machines. The helicity of a particular conformer of the molecular rotary motor induces a helical superstructure in the liquid crystal host, which can in turn result in a remarkably high degree of circular polarization of the bright photoluminescence from achiral BODIPY dyes dispersed in the liquid crystal host (with quantum yield *φ*_PL_ up to 0.75 and |*g*_lum_| up to 0.45). The circular polarization can non‐invasively be programmed via external stimuli i.e., light irradiation. The contrast in circular polarization that can be induced between different parts of the active layer is large enough to be observed by the naked eye. The versatility i.e. tunability of our approach follows from the possibility to combine various machines and dyes into the LC host material without the need for any chemical modification of the components in the mixture. The persistence of the photoprogrammed polarization can be varied by engineering the relative stability of states of the particular molecular machine used, while the color of the luminescence can be tuned by varying the type of dye employed. Our findings may enable combination of the responsive photoluminescent layers presented here with multicolor light emitting diodes to fabricate pixilated displays with externally addressable polarization. The prospects for new opportunities to realize 3D stereographic displays seems bright.

## Conflict of interest

The authors declare no conflict of interest.

1

## Supporting information

As a service to our authors and readers, this journal provides supporting information supplied by the authors. Such materials are peer reviewed and may be re‐organized for online delivery, but are not copy‐edited or typeset. Technical support issues arising from supporting information (other than missing files) should be addressed to the authors.

Supporting Information

## Data Availability

The data that support the findings of this study are available in the supplementary material of this article.
