# Supplementary material for: Programming and Dynamic Control of the Circular Polarization of Luminescence from an Achiral Fluorescent Dye in a Liquid Crystal Host by Molecular Motors
Source: Angew Chem Int Ed Engl. 2022 Sep 5;61(41):e202206310. doi: 10.1002/anie.202206310 (PMC9826132; doi:10.1002/anie.202206310)
Supplement: Supplementary file 1 — Supporting Information [file ANIE-61-0-s001.pdf]

Angewandte Chemie
Eine Zeitschrift der Gesellschaft Deutscher Chemiker

Supporting Information

Programming and Dynamic Control of the Circular Polarization of Luminescence from an Achiral Fluorescent Dye in a Liquid Crystal Host by Molecular Motors

J. Hou, R. Toyoda, S. C. J. Meskers*, B. L. Feringa**

Supporting Information

Materials and methods

All chemicals and solvents (analytical grade) were purchased from Tokyo Chemical Industry Co. Ltd., Sigma-Aldrich Co. LLC, or Thermo Fisher Scientific Inc., unless otherwise stated. **BG**, **OB**, **BB**, **M1**, **M2**, **M3**, and **M4** were prepared according to the published procedures. [1-8] ^{1}H and ^{13}C nuclear magnetic resonance (NMR) data were collected in $CDCl_3$ and recorded on a Varian Mercury-Plus 400 or a Bruker Avance 600 NMR spectrometer at 298 K unless indicated otherwise. Chemical shifts are given in parts per million (ppm) relative to the residual solvent signal. Multiplets in ^{1}H NMR spectra are designated as follows: s (singlet), d (doublet), t (triplet), q (quartet), m (multiplet), br (broad). High resolution mass spectrometry was performed on an LTQ Orbitrap XL spectrometer. Enantiopure switches and motors were separated with chiral HPLC or SFC. **M1** was separated with HPLC. (Chiralcel-ODH, eluent: heptane/isopropyl alcohol = 99.7/0.3). **M2** was separated with HPLC (Chiralcel-ODH). **M3** was separated with HPLC. (Chiralpak-ADH, eluent: heptane/isopropyl alcohol = 99/1). **M4** was separated with HPLC. (Chiralcel-ODH, eluent: 100 % heptane). Wedge cells for Grandjean-Cano wedge method (KCRK-07, $\tan\theta$=0.0785) were provided by Japan EHC Co., Ltd. To induce planar anchoring, a quartz substrate was thoroughly cleaned with water, acetone and ethanol, and spin-coated with polyvinyl alcohol layer. The coated substrate was rubbed with velvet in a certain direction. Two of the substrates were put together by the UV-curing spacer to construct a LC cell with the spacing distance varying

from 3 to 25 µm. UV/vis absorption spectra were obtained with an Agilent 8453 UV-vis Diode Array System at room temperature. Circular dichroism (CD) spectra were recorded on a JASCO J-715 or a JASCO 810 spectropolarimeter at room temperature. Results are plotted as circular differential absorbance A_L-A_R, i.e. the difference between the absorbances for left and right-circularly polarized light. The circular differential absorbance, a dimensionless quantity also abbreviated as ΔA, is proportional to the ellipticity θ in millidegrees via: $\Delta A = \theta\,[\mathrm{mdeg}]/33000$.[9] Fluorescence spectra were collected with a JASCO FP-6200 spectrometer. Generalized ellipsometry was performed on a VASE instrument from Woollam. Irradiation experiments were performed using either LED lamps (365 nm, 405 nm, 455 nm, 470 nm, 530 nm and 617 nm) obtained from Thorlabs Inc or a handhold LED lamp (312 nm). All optical phenomena of the LC mixtures were observed and recorded via a polarized optical microscope (POM, DM2700p, Leica).

CPL measurement

Circular Polarization of Luminescence measurements were performed using a home-built spectrometer, involving photoelastic modulation at 50 kHz, parallel multichannel detection and single photon counting electronics.[10] The instrument is capable of measuring both circular and linear polarizations. In the polarization measurement, the emission collection and photoexcitation were performed in a direction perpendicular to the luminescent layer and with an in-line geometry. Excitation light was depolarized by sending it through an optical fiber.

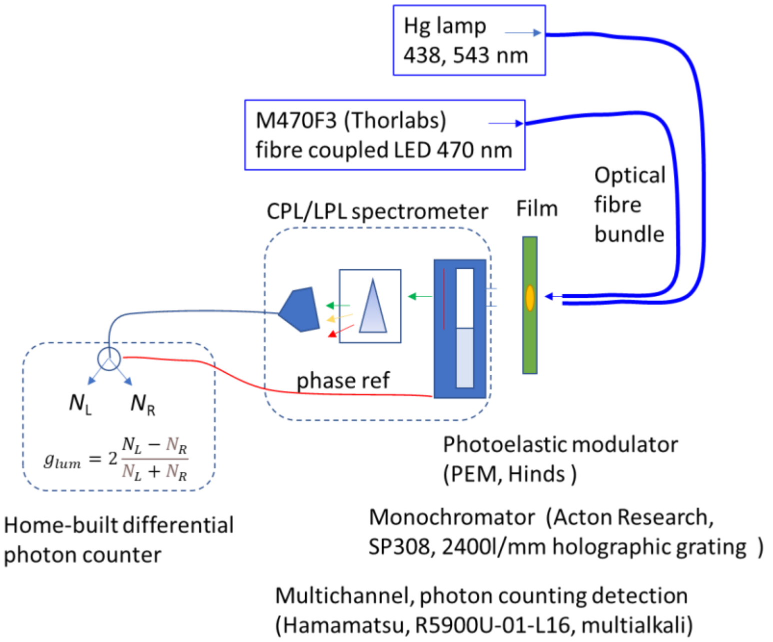

Figure S1. A Schematic representation of experimental setup for CPL measurement of film samples.

Synthesis of RB:

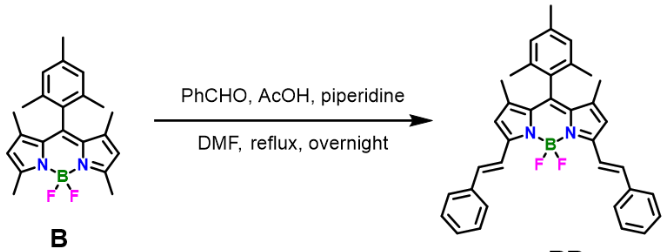

To a DMF solution (15 mL) of **B** (200 mg, 0.55 μmol), benzaldehyde (0.53 mL, 5.1 μmol), acetic acid (1 mL) and piperidine (1 mL) were added and the mixture was heated at reflux overnight. After cooling to room temperature, the mixture was diluted with water and extracted with ethyl acetate. The organic layer was washed with brine and dried over $MgSO_4$. The solvent was evaporated under reduced pressure and the crude product was purified by column chromatography (SiO_2, DCM:pentane = 1:4, v/v). Reprecipitation from DCM/hexane gave **RB** as a blue powder (101 mg, 0.19 μmol, 34%).

^{1}H NMR (600 MHz, Chloroform-*d*) δ 7.76 (d, *J* = 16.3 Hz, 1H), 7.64 (d, *J* = 7.6 Hz, 2H), 7.41 (dd, *J* = 7.6 Hz, 2H), 7.32 (dd, *J* = 7.3 Hz, 1H), 7.26 (d, *J* = 16.3 Hz, 1H), 6.98 (s, 1H), 6.64 (s, 1H), 2.36 (s, 1H), 2.13 (s, 3H); ^{13}C NMR (151 MHz, Chloroform-*d*) δ 152.58, 141.63, 139.22, 138.85, 136.82, 136.12, 135.48, 132.74, 131.32, 129.11, 128.99, 128.92, 127.66, 119.55, 117.61, 21.37, 19.82, 13.81; HRMS (APCI pos) *m/z* calcd for $C_{36}H_{33}N_2BF_2$ $[M]^+$ 542.2699, found 542.2690.

Sample preparation

BODIPY (**BB**, **GB**, **OB** or **RB**), molecular machine (**M1**, **M2**, **M3** or **M4**) and LC mixture (E7 or ZLI-1132) were dissolved together in DCM and stirred at 60 °C until DCM was removed. The resulted mixture was filled into LC cells at 60 °C by capillary suction. Samples were allowed to cool down to room temperature after filling.

Table S1. Compositions of **MCELD**s.

	Molecular Machine	BODIPY (0.04 wt%)	Liquid Crystal
MCELD-1	2 wt% M1	GB	E7
MCELD-2	2 wt% M1	OB	E7
MCELD-3	2 wt% M1	RB	E7
MCELD-4	1 wt% M2	BB	ZLI-132
MCELD-5	1 wt% M3	GB	E7
MCELD-6	1 wt% M4	GB	E7

HTP measurement

Helical twisting power (HTP) of molecular machines and the changes in HTP upon light irradiation were determined by the Grandjean-Cano method.[11] HTP (β) is defined as follows: $\beta = 1 / (pc)$, where p is the helical pitch and c is the molar or mass concentration. The pitch is determined by: $p = 2R \tan\theta$, where R represents the distance between the disclination lines and θ is the wedge angle of the wedge cells ($\tan \theta = 0.00785$). A liquid crystal mixture was prepared by doping an enantiopure molecular machine into LC, and then it was filled into the wedge cell by capillary force. The wedge cell was heated to 60 °C then cooled down to room temperature with a cooling rate of -1 °C·min^{-1}. The disclination lines were observed through POM, which is a characteristic of cholesteric organization of liquid crystal molecules. The length of R was determined by measuring the intervals between the disclination lines.

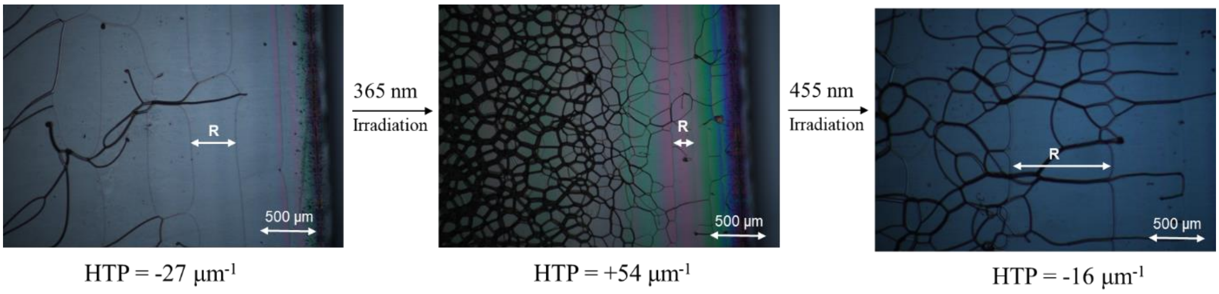

Figure S2. POM images of stripe wedge Grandjean Cano cell filled with 1 wt % (*S*)-**M1** in E7 before and after 365 nm and 455 nm light irradiation.

Table S2. Helical twisting powers of **M1**, **M2**, **M3** and **M4** at different states.

Helical twisting power (in wt%)

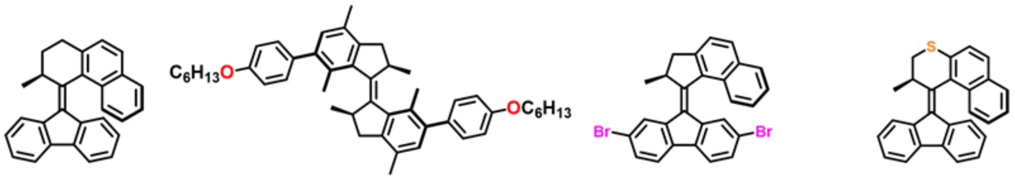

M1 M2 M3 M4

	$HTP_{wt\%}$(Initial)	$HTP_{wt\%}$(PSS1)	$HTP_{wt\%}$(PSS2)
**M1**	-27 μm^{-1}	+ 54 μm^{-1} (365 nm)	-16 μm^{-1} (455 nm)
**M2**	+ 75.5 μm^{-1} (cis)	-88.9 μm^{-1} (312nm)	+4.8 μm^{-1} (365 nm)
**M3**	-115 μm^{-1}	+97 μm^{-1} (365 nm)	-107 μm^{-1} (THI)
**M4**	-8 μm^{-1}	+ 54 μm^{-1} (365 nm)	+17 μm^{-1} (455 nm)

Photo luminescent quantum yield (PLQY) measurement

Background correction was done by subtracting the dark spectra from the corresponding reference or sample measurement spectrum. Then QY calculation was done according to the two-measurement approach as described by Leyre et al.[12]

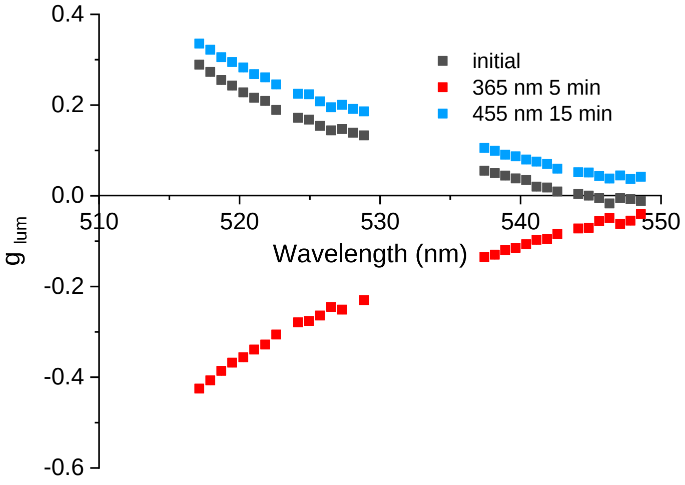

Figure S3. CPL spectral change of a device with 2 wt% (*R*)-**M1** and 0.04 wt% **GB** in E7 prepared in a 25 μm-thick LC cell. Initial state (black), 365 nm PSS (red) and 455 nm PSS (blue). Excited at 470 nm.

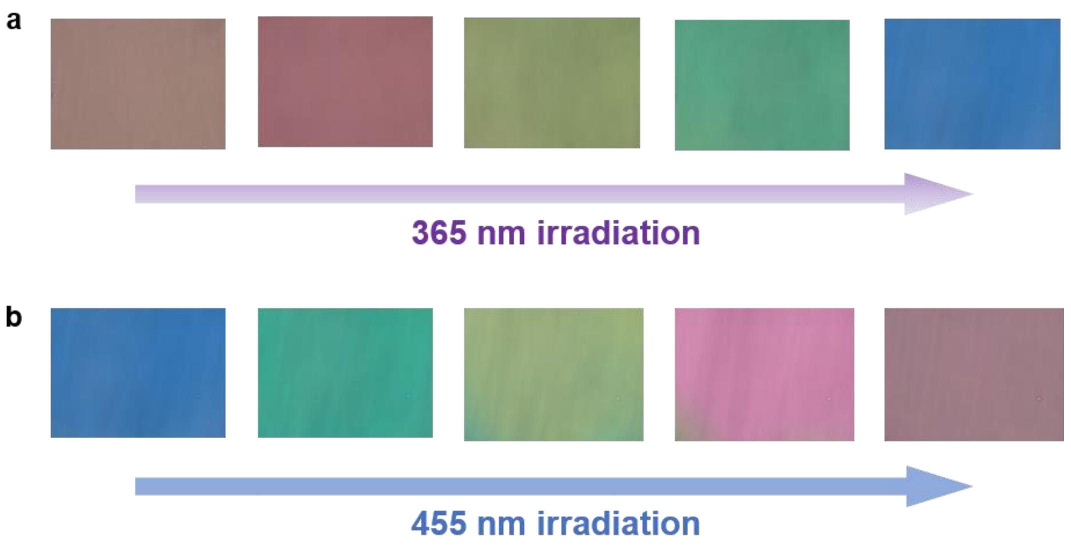

Figure S4. POM images showing reflection color change of a device with 1 wt% (*S*)-**M1** in E7 prepared in a 10 μm-thick LC cell upon (a) 365 nm irradiation and (b) 455 nm irradiation.

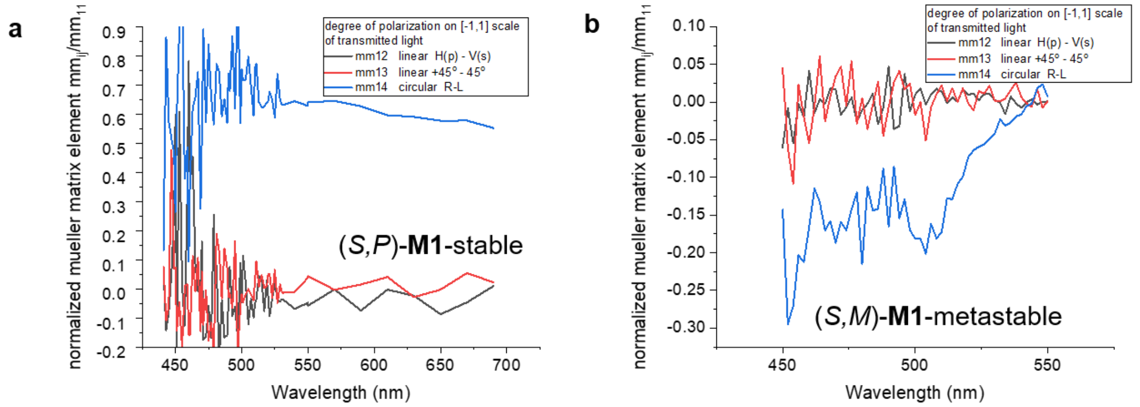

Figure S5. Generalized ellipsometry spectra of a device with 2 wt% (*S*)-**M1** and 0.04 wt% **GB** in E7 prepared in a 25 μm-thick LC cell (a) at the initial state and (b) after 365 nm irradiation. Shown are the spectra of some Mueller matrix elements normalized to the total transmitted intensity for unpolarized light at normal incidence ; black : mm_{12} (linear differential transmission , $mm_{12} = (I_H - I_V) / (I_H + I_V)$ with $I_{H(V)}$ the total transmitted intensity for horizontallly (vertically) polarized incident light; red : mm_{13} (linear differential transmission; $mm_{13} = (I_{+45} - I_{-45}) / (I_{+45} + I_{-45})$ with $I_{+45(-45)}$ the total transmitted intensity for incident light linearly polarized in the direction at +45° (-45°) relative to the vertical; blue: mm_{14} (circular differential transmission; $mm_{14} = (I_R - I_L) / (I_R + I_L)$ with $I_{R(L)}$ the total transmitted intensity for right (left) circularly polarized incident light .

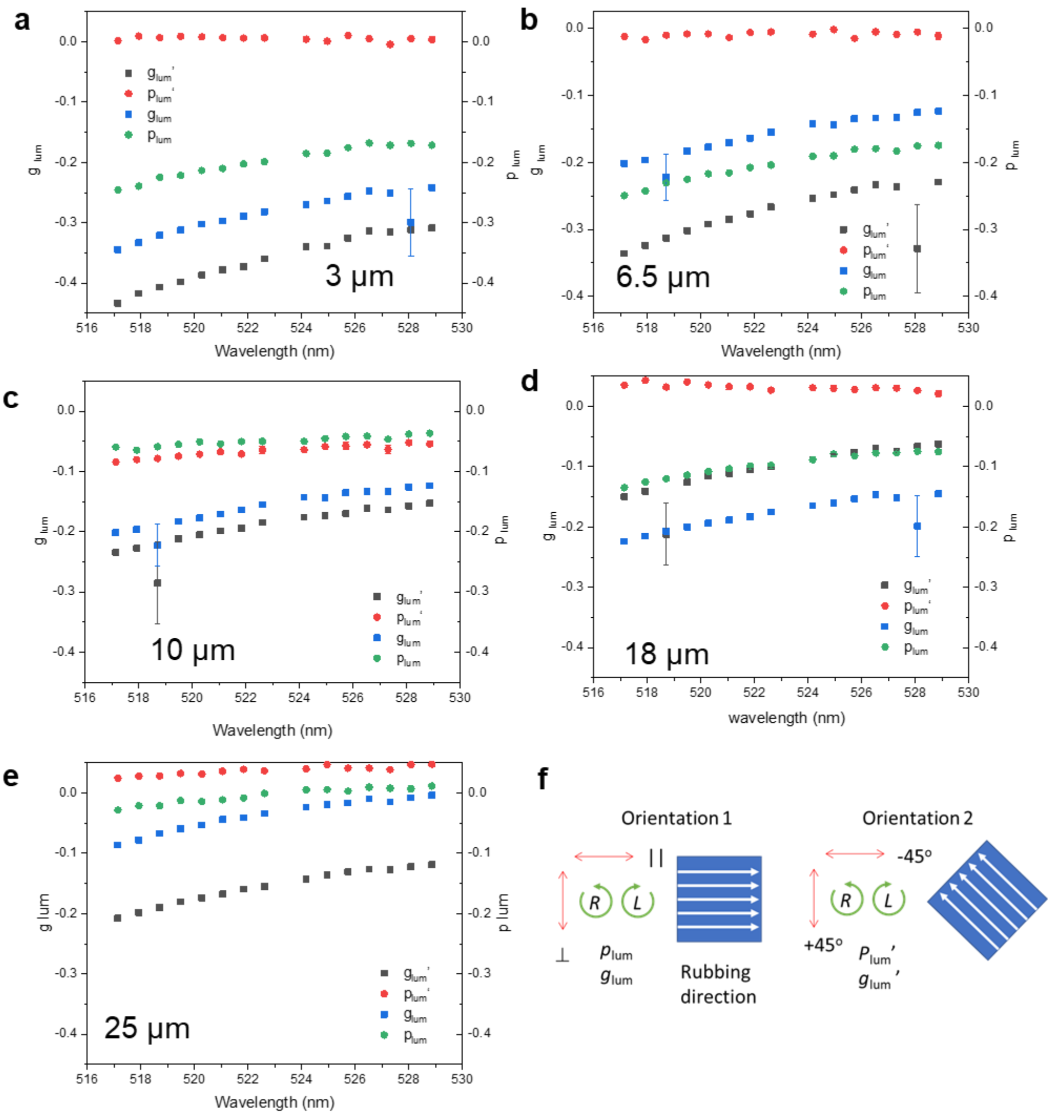

Figure S6. CPL (grey and blue) and LPL (red and green) spectra of devices with 1.5 wt% (*S*)-**M1** and 0.04 wt% **GB** in E7 prepared in LC cells with different thicknesses: (a) 3 μm, (b) 6.5 μm, (c) 10 μm, (d) 18 μm and (e) 25 μm. (f) The spectra were taken for two different orientations of the films relative to the orientation of the photoelastic modulator. In the first orientation, the polarization directions for the two independent measurements of the intensities of linearly polarized light are parallel and perpendicular to the alignment direction of the cell yielding $p_{lum} = 2\frac{I_{||}-I_{\perp}}{I_{||}+I_{\perp}}$. The corresponding degree of circular polarization in this orientation is g_{lum}. In the second orientation, the polarization directions make an angle of 45° with the rubbing direction of the cell yielding $p_{lum}' = 2\frac{I_{+45}-I_{-45}}{I_{+45}+I_{-45}}$ and g_{lum}'. We note that g_{lum} and g_{lum}' are not fully identical. The difference can be diminished when the alignment layer was removed. Wavelength of excitation light used: 470 nm. We note that for all datapoints in graphs (a-e), error margins have been included, yet for almost all measurements, the error margins are too small to be visible behind the symbol used for representing the datapoint ($\sigma < 10^{-2}$).

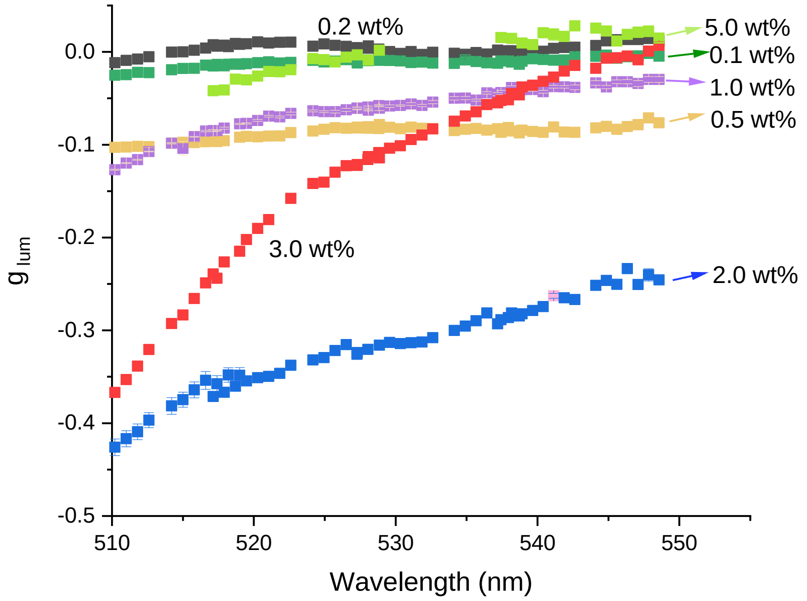

Figure S7. CPL spectra of devices with (*S*)-**M1** (0.1, 0.2, 0.5, 1.0, 2.0, 3.0, 5.0 wt%) and 0.04 wt% **GB** in E7 prepared in 25 μm-thick LC cells. Excited at 470 nm.

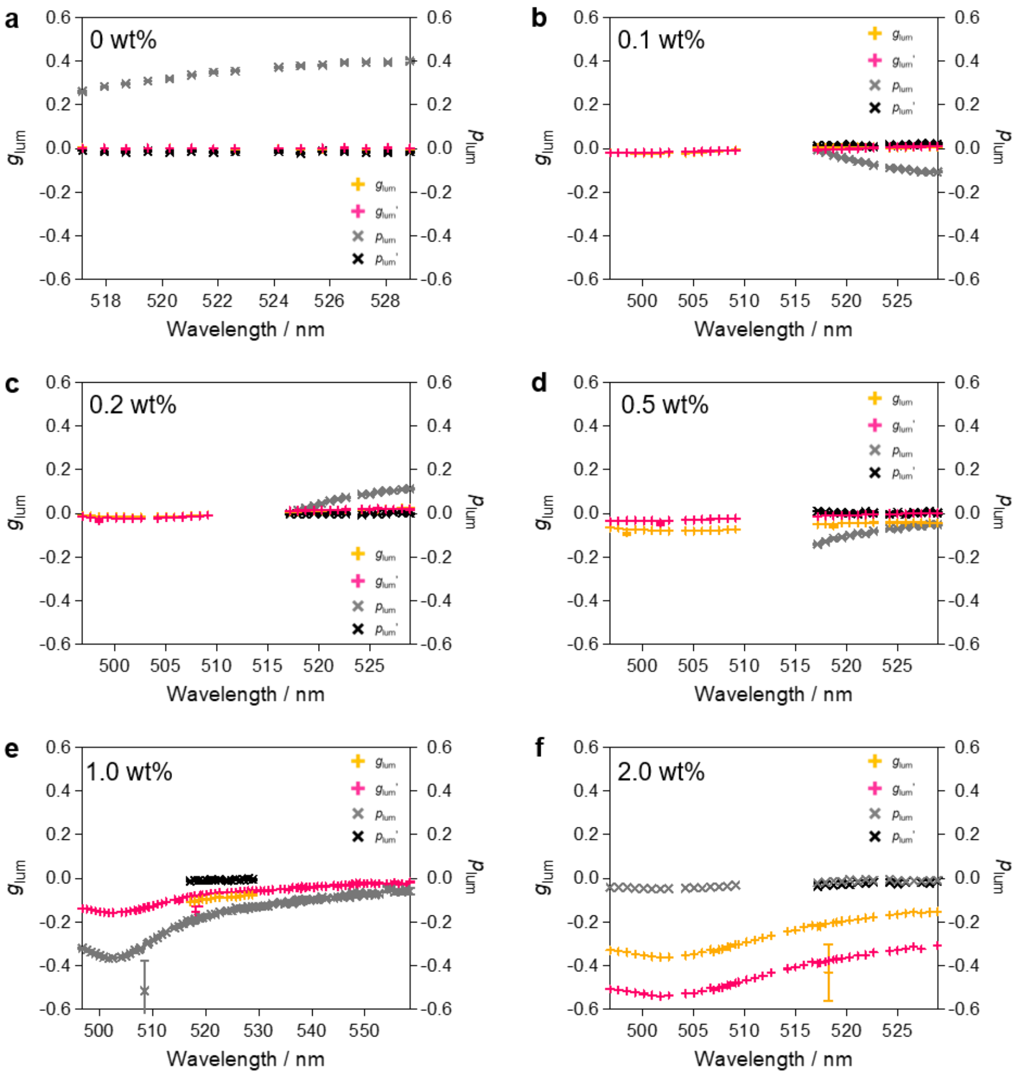

Figure S8. CPL and LPL spectra of devices with (*S*)-**M1** (varying concentration from 0.1 to 2.0 wt%) and 0.04 wt% **GB** in E7 prepared in 25 µm-thick LC cells. (a) 0 wt %. (b) 0.1 wt %. (c) 0.2 wt %. (d) 0.5 wt%. (e) 1.0 wt%. (f) 2.0 wt%. The spectra were taken for two different orientations of the films relative to the orientation of the photoelastic modulator. In the first orientation, the polarization directions for the two independent measurements of the intensities of linearly polarized light are parallel and perpendicular to the alignment direction of the cell yielding $p_{lum} = 2\frac{I_{||}-I_{\perp}}{I_{||}+I_{\perp}}$. The corresponding degree of circular polarization in this orientation is g_{lum}. In the second orientation, the polarization directions make an angle of 45° with the rubbing direction of the cell yielding $p_{lum}' = 2\frac{I_{+45}-I_{-45}}{I_{+45}+I_{-45}}$ and g_{lum}'. Excitation wavelength at 470 nm.

Effect of circular dichroic self-absorption on the circular polarization of luminescence.

For organic fluorescent dyes there is almost invariable always some overlap between the fluorescence ($S_1 \rightarrow S_0$) and the lowest absorption band ($S_1 \leftarrow S_0$). This implies that some of the photons emitted as fluorescence will be reabsorbed by (other) molecules in the solution or matrix. For solutions of chiral molecules, the probabilities for absorption of left and right circular polarized photons are not the same, and this circular selective self-absorption may contribute in an artificial manner to the circular polarization of luminescence.

To estimate the effect of circular dichroic self-absorption on the apparent degree of circular polarization of luminescence g_{lum} , we start from the definition of the dissymmetry factor

$$g_{lum} = 2\frac{I_L - I_R}{I_L + I_R}$$

We then assume, for the moment, that all circular polarization in luminescence is the result of selective absorption of one circular polarizations of light by the sample itself. In such an extreme case the intensities of left and right circularly polarized emission light may be approximated by

$$I_L = I_0 10^{-A_L};\ I_R = I_0 10^{-A_R}$$

Where A_L and A_R denote the absorbance for left and right circularly polarized light of the entire film. Here it is implicitly assumed that the luminescence is generated in a thin layer at the side of the film facing the excitation source. Adopting this worst case scenario, we then estimate the artificial degree of circular polarization due to Circular Dichroic Self-Absorption ($g_{\text{lum}}^{\text{CDSA}}$) as :

$$g_{lum}^{CDSA} = 2\frac{10^{-A_L} - 10^{-A_R}}{10^{-A_L} + 10^{-A_R}}$$

In the case where the absorbances are small (A <<1), we can develop the exponential function in a Taylor series and retain only the lowest order terms :

$$10^{-A_L} \cong 1 - \ln(10)\, A_L + \cdots$$

This then directly relates the artificially induced dissymmetry ratio to the circular dichroism of the film:

$$g_{lum}^{CDSA} \cong -\ln(10)(A_L - A_R)$$

Looking now at Figure 3b, we find that, in the initial state at 520 nm (A_L - A_R) = -0.01. Then circular dichroic self-absorption alone is expected to give rise to a dissymmetry factor $g_{\text{lum}}^{\text{CDSA}} \approx +0.03$. The actual experiment (Figure 2) yields $g_{\text{lum}} \approx -0.4$. Hence both sign and magnitude of the experimental g_{lum} value in comparison to the circular dichroism, argue against a major contribution from circular dichroic self-absorption to the circular polarization of luminescence.

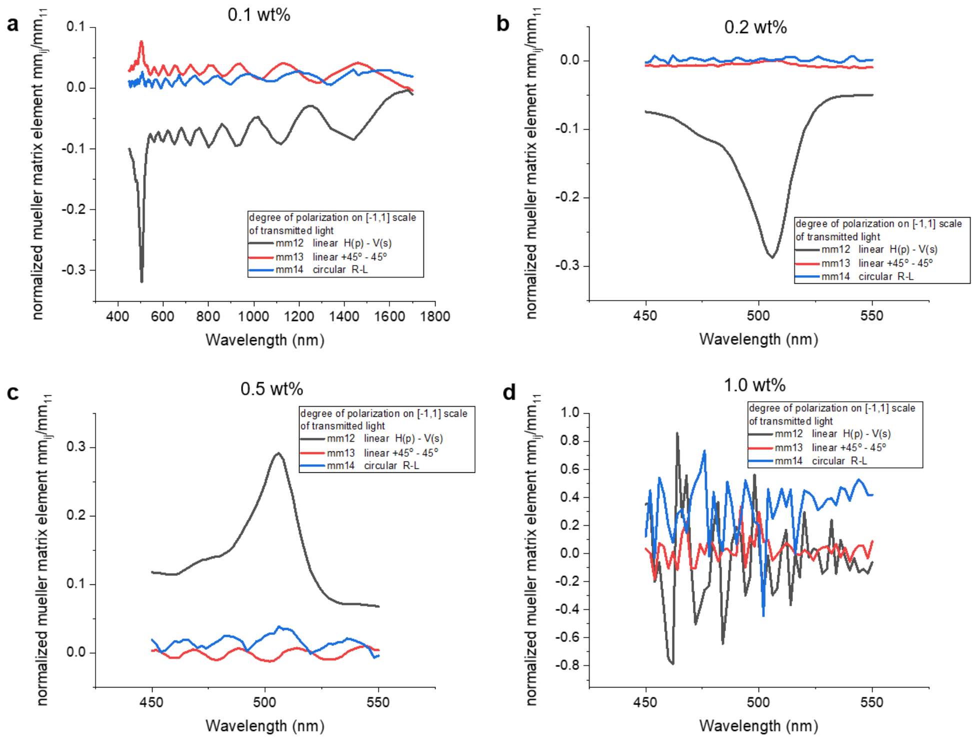

Figure S9. Generalized **e**llipsometry spectra of devices with (*S*)-**M1** (varying concentration from 0.1 to 1.0 wt%) and 0.04 wt% **GB** in E7 prepared in 25 μm-thick LC cells. (a) 0.1 wt %. (b) 0.2 wt %. (c) 0.5 wt %. (d) 1.0 wt %. Shown are the spectra of some mueller matrix elements normalized to the total transmitted intensity for unpolarized light at normal incidence ; mm_{12} (black), mm13 (red), mm_{14} (blue).

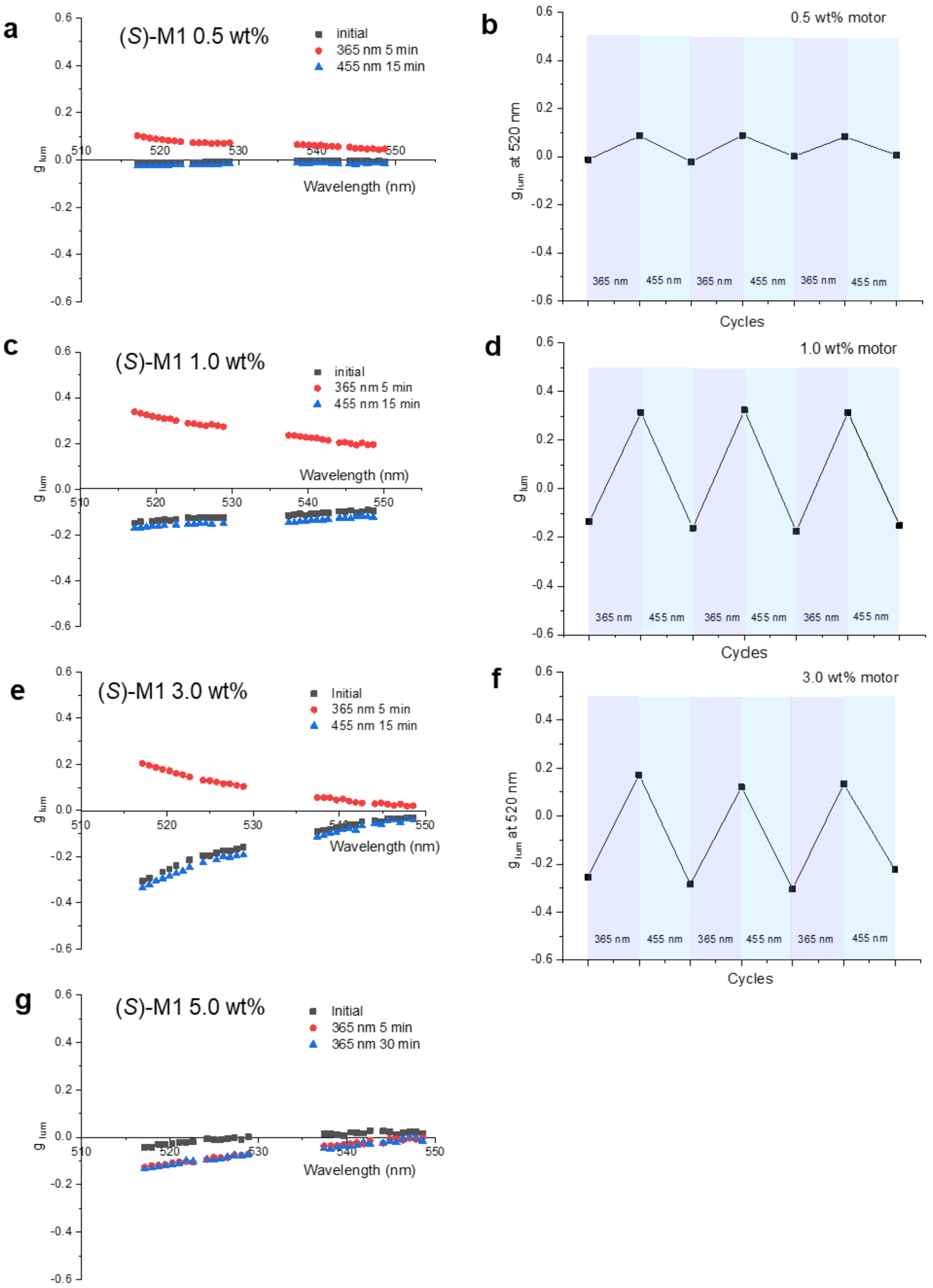

Figure S10. CPL spectral control with 365 nm and 455 nm light. CPL spectral change of devices (a) with 0.5 wt % (*S*)-**M1**, (c) with 1.0 wt % (*S*)-**M1**, and (e) with 3.0 wt% (*S*)-**M1**. (b,d,f) The irradiation cycle was repeated for three times for each device. All the devices are prepared in 25 μm-thick LC cells and include 0.04 wt% **GB** in E7. Excited at 470 nm.

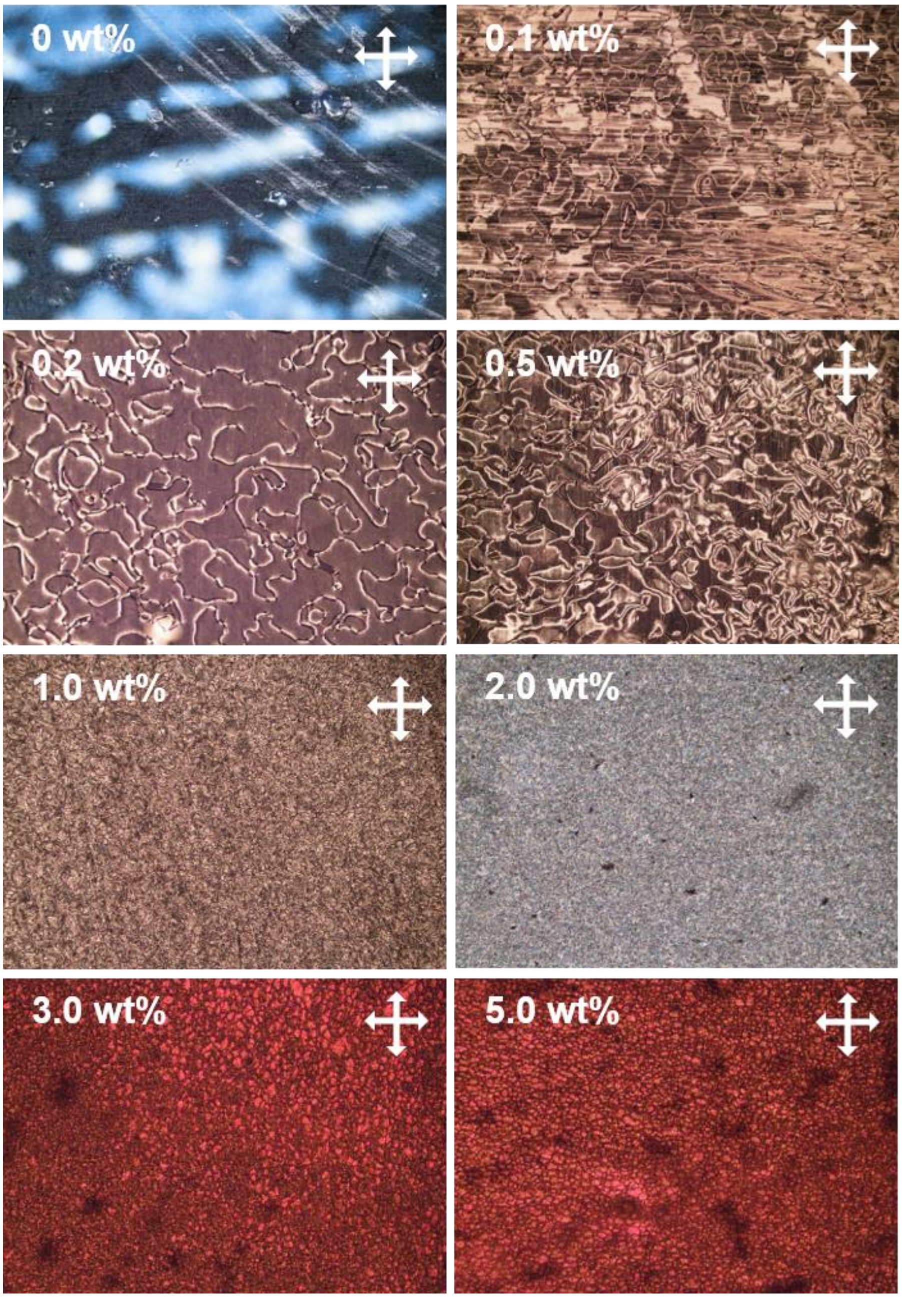

Figure S11. POM images of devices with varied (*S*)-**M1** concentration (0 to 5.0 wt%). All the devices are prepared in 25 μm-thick LC cells and include 0.04 wt% **GB** in E7. The crossed arrows indicate the direction of the polarizers.

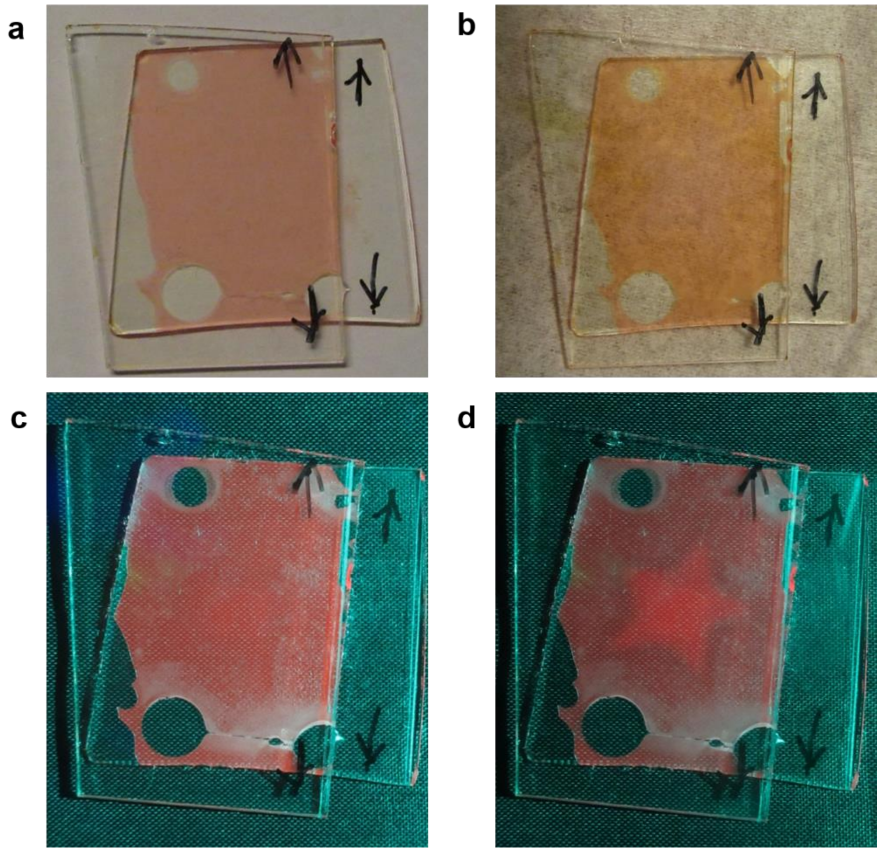

Figure S12. Pictures of a device with 2.0 wt% (*S*)-**M1** and 0.04 wt % **OB** in E7 prepared in a 10 µm-thick LC cell. (a) Before irradiation. After 365 nm irradiation with a star-shaped mask (b) under ambient light, (c) under 470 nm light, and (d) under 470 nm light through a circular polarizer. For (c) and (d), a longpass filter was used to cut off light with wavelength shorter than 500 nm.

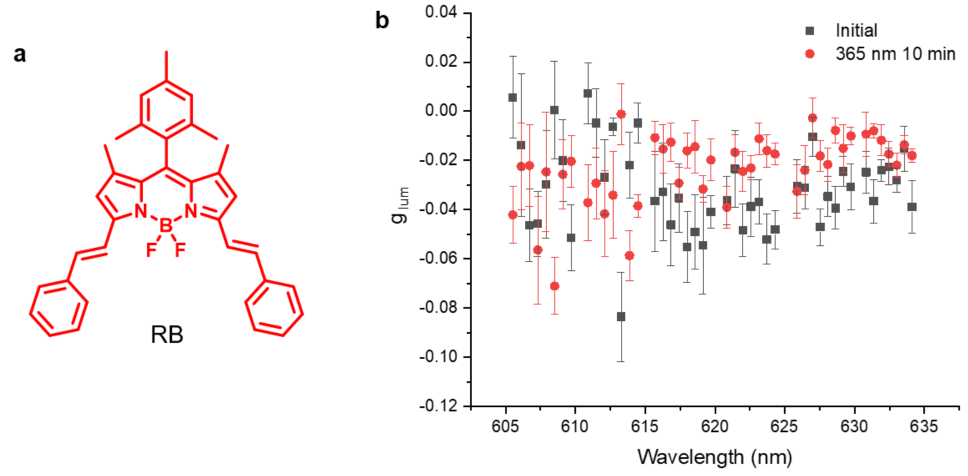

Figure S13. CPL of a device with 2.0 wt% (*S*)-**M1** and 0.04 wt% **RB** in E7 prepared in a 25 μm-thick LC cell. (a) Chemical structure of **RB**. (b) CPL spectra of the device at the initial state (black) and after 365 nm irradiation (red). Excited at 543 nm.

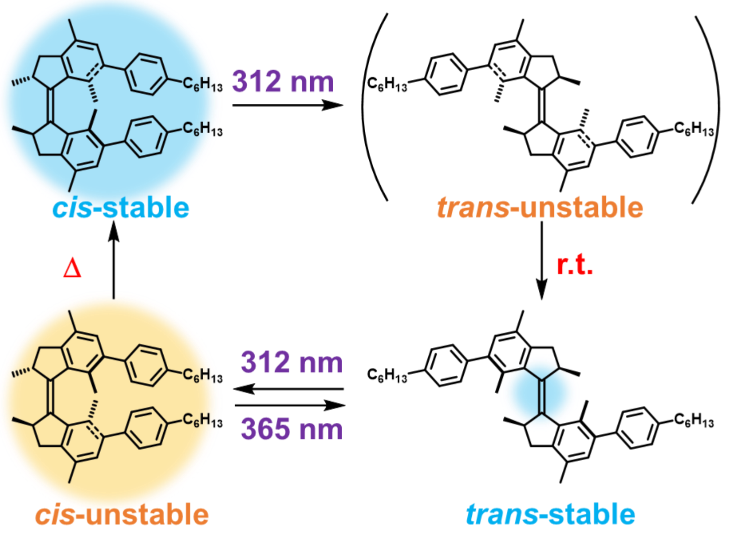

Figure S14. Photo-responsive molecular motor (*S*)-**M2** and its full rotation cycle.

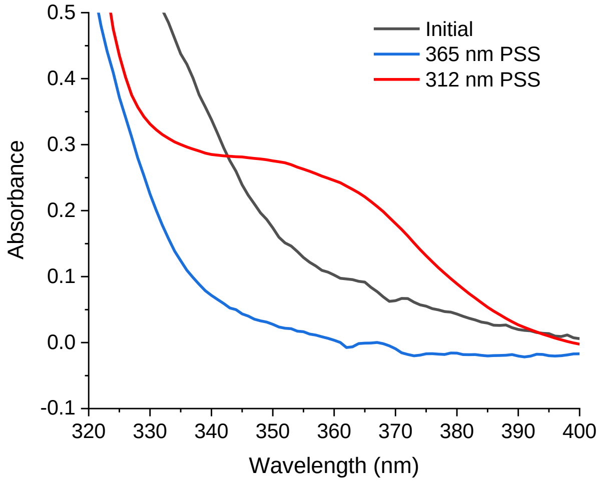

Figure S15. UV/vis spectra of a device with 1 wt% (*S*)-**M2** in ZLI-1132 prepared in a 25 μm-thick LC cell before irradiation (black), after 312 nm irradiation until no further change was observed (red) and after irradiation back to the *trans*-stable state with 365 nm for 30 min (blue).

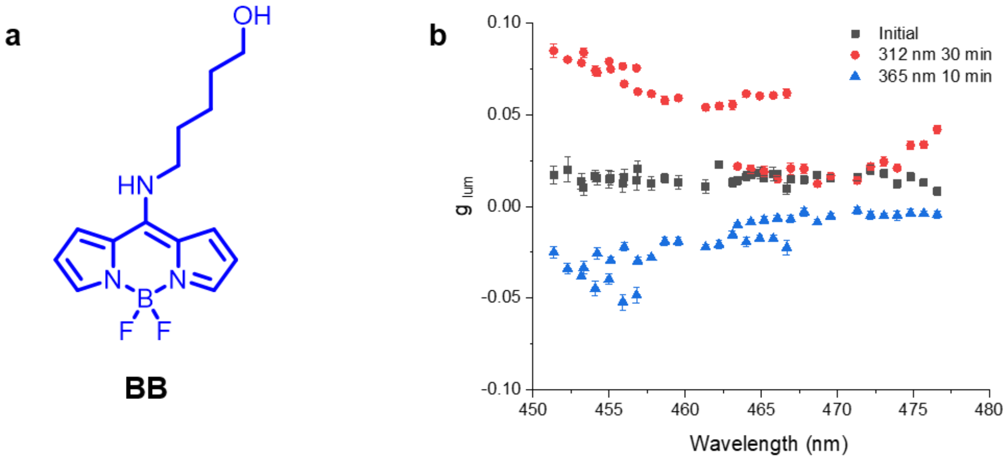

Figure S16. CPL of a device with 1.0 wt% (*S*)-**M2** and 0.04 wt% of **BB** in ZLI-1132 prepared in a 25 μm-thick LC cell. (a) Chemical structure of **BB**. (b) CPL spectra of the device at the initial state (black) and after irradiation with 312 nm light (red) and 365 nm light (blue). Excited at 438 nm.

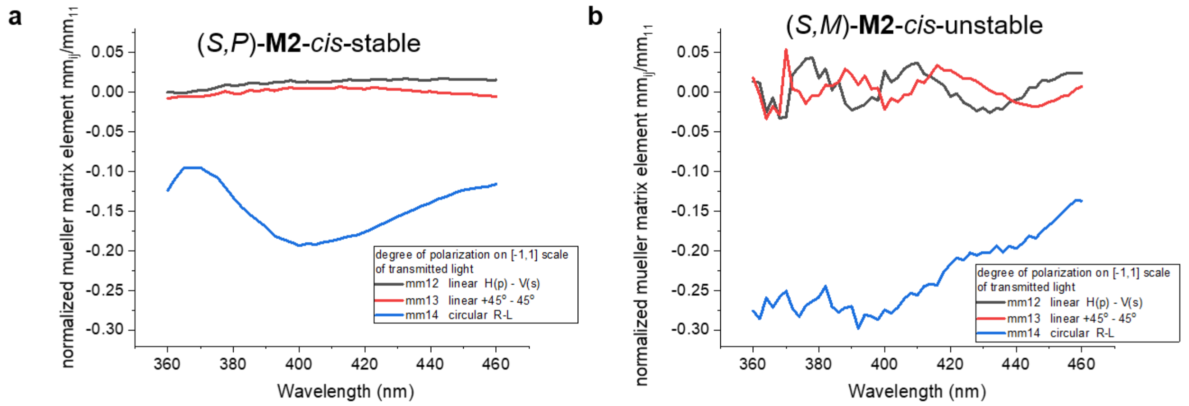

Figure S17. Generalized ellipsometry spectra of a device with 1.0 wt % (*S*)-**M2** and 0.04 wt % **BB** in ZLI-1132 prepared in a 25 μm-thick LC cell (a) at the initial state and (b) after 312 nm irradiation for 30 min. Shown are the spectra of some mueller matrix elements normalized to the total transmitted intensity for unpolarized light at normal incidence; mm_{12} (black), mm13 (red), mm_{14} (blue).

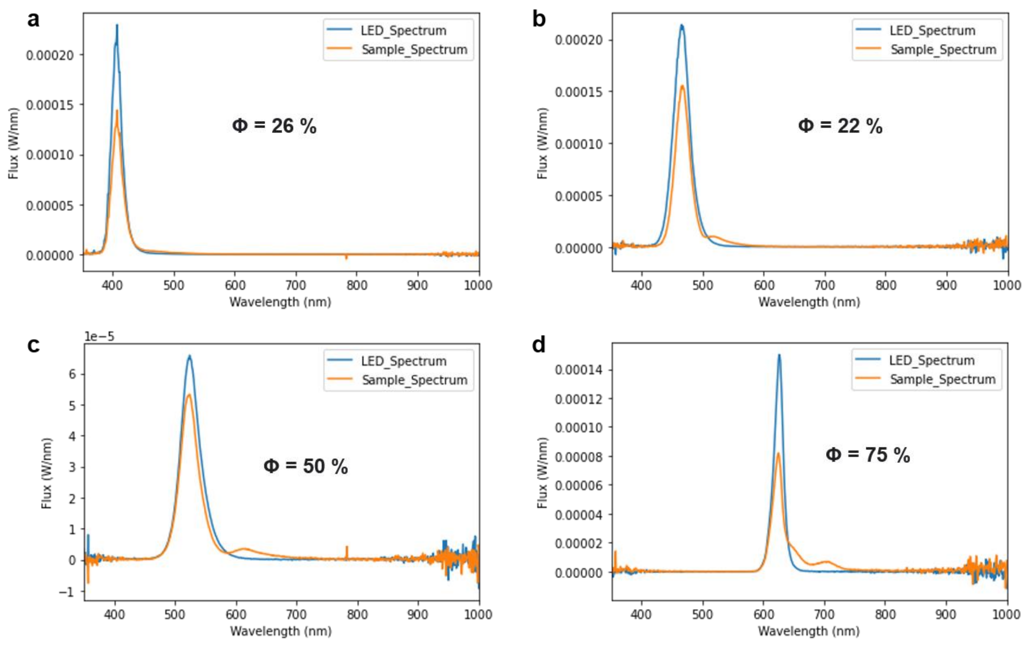

Figure S18. Quantum yields of devices with (a) 1.0 wt % (*S*)-**M2** and 0.04 wt% of **BB** prepared in ZLI-1132 prepared in a 25 µm-thick LC cell (Illumination source: 405 nm LED), (b) 2.0 wt% (*S*)-**M1** and 0.04 wt% **GB** in E7 prepared in a 25 µm-thick LC cell (Illumination source: 470nm LED), (c) 2.0 wt% (*S*)-**M1** and 0.04 wt% of **OB** in E7 prepared in a 25 µm-thick LC cell (Illumination source: 530 nm LED) and (d) 2.0 wt% (*S*)-**M1** and 0.04 wt% of **RB** in E7 prepared in a 25 µm-thick LC cell (Illumination source: 617 nm LED). All samples were measured 3 times, respectively.

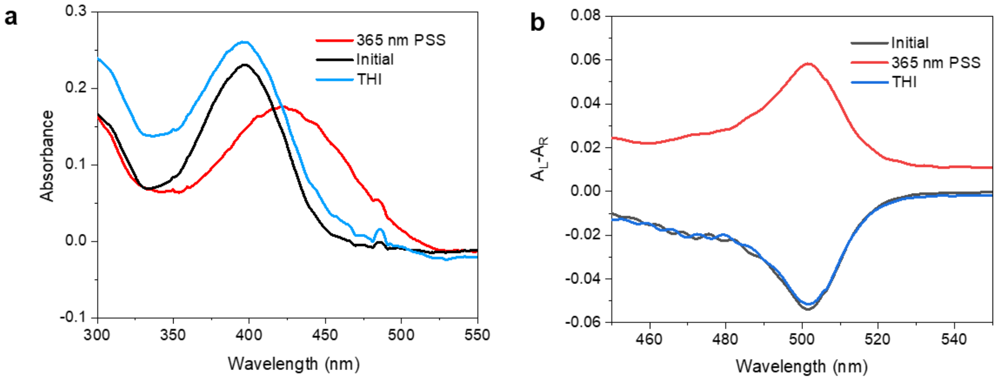

Figure S19. (a) UV/vis absorption spectra and (b) CD spectra of a device with 1.0 wt% (*S*)-**M3** and 0.04 wt% of **GB** in E7 prepared in a 10 µm-thick LC cell at the initial state (black), at 365 nm PSS (red) and after thermal helix inversion at 25 °C (blue).

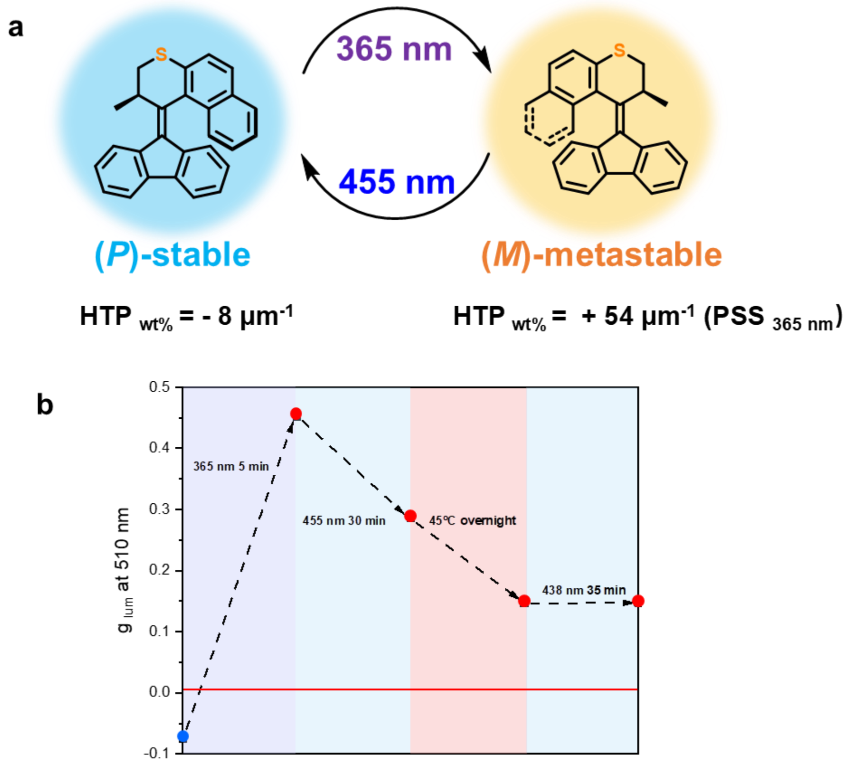

Figure S20. CPL switching of a device with 1 wt% (*S*)-**M4** and 0.04 wt% **GB** in E7 prepared in a 25 μm-thick LC cell. (a) Photo-responsive molecular chiral switch (*S*)-**M4**. (b) CPL spectral change upon 365 nm and 455 nm irradiation, 45 °C heating and 438 nm irradiation. Excited at 470 nm.

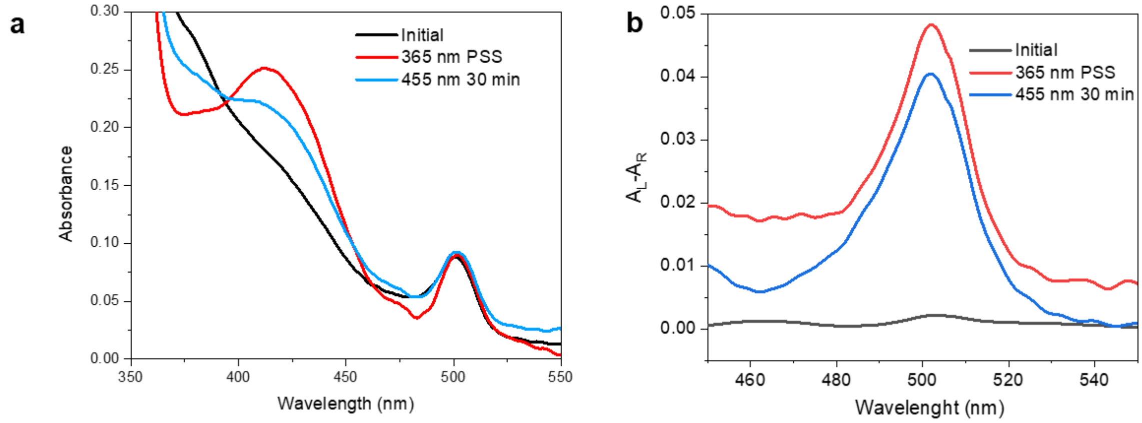

Figure S21. (a) UV/vis absorption spectra and (b) circular dichroism spectra of a device with 1 wt% (*S*)-**M4** and 0.04 wt% **GB** in E7 prepared in a 10 μm-thick LC cell at the initial state (black), at 365 nm PSS (red) and after 455 nm light irradiation for 30 min (blue).

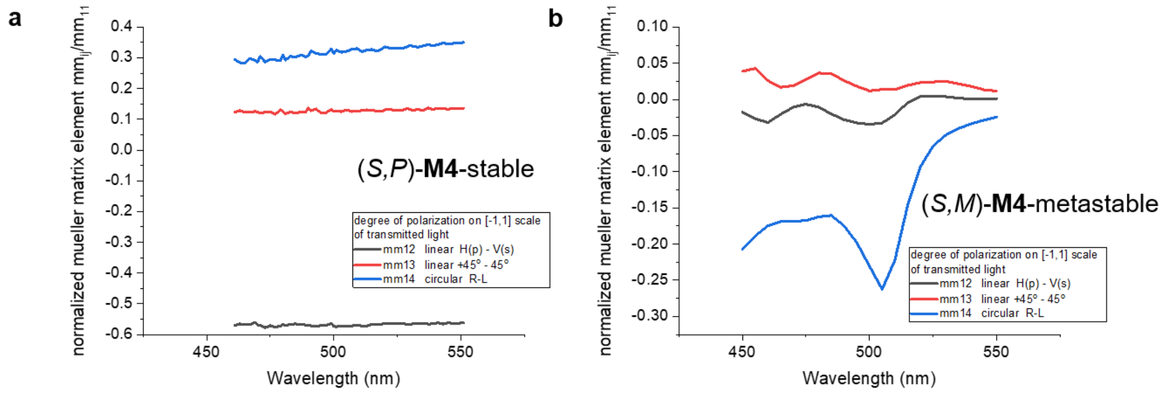

Figure S22. Generalized ellipsometry spectra of a device with 1 wt% (*S*)-**M4** and 0.04 wt% **GB** in E7 prepared in a 25 μm-thick LC cell (a) at the initial state and (b) after 365 nm irradiation. Shown are the spectra of some mueller matrix elements normalized to the total transmitted intensity for unpolarized light at normal incidence; mm_{12} (black), mm13 (red), mm_{14} (blue).

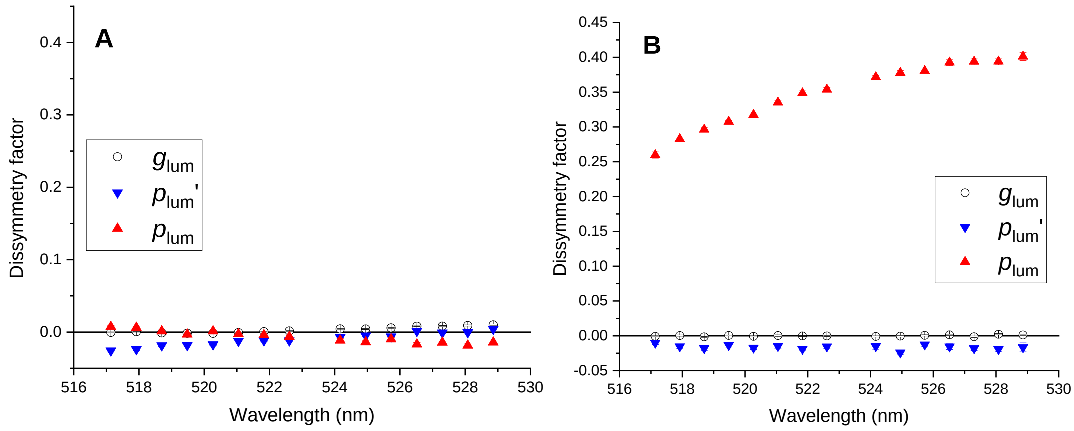

Figures S23. CPL and LPL spectra of control samples. **A**: control experiment, 0.04wt% **GB** in E7 liquid crystal, 25 μm thickness, no alignment layers, no chiral dopant, excitation at 470 nm wavelength. All dissymmetry factors g_{lum}, p_{lum}, p_{lum}' are very small. **B**: control experiment, 0.04wt% **GB** in E7 liquid crystal, with alignment layers but no chiral dopant, excitation at 470 nm wavelength. The dissymmetry factors g_{lum}, p_{lum}' are very small. In contrast, p_{lum}, the dissymmetry factor for light with linear polarization parallel and perpendicular to the rubbing direction is large, indicating alignment of the transition dipole moment of the dye parallel to the rubbing direction.

References

1. A. B. Nepomnyashchii, M. Bröring, J. Ahrens, A. J. Bard. *J. Am. Chem. Soc.* **2011**, 133, 8633–8645

2. L. Bonardi, G. Ulrich, R. Ziessel. *Org. Lett.* **2008**, 10, 2183–2186

3. D. Kim, *et al. J. Fluoresc.* **2017**, 27, 2231–2238

4. J. Vicario, A. Meetsma, B. L. Feringa, *ChemComm* **2005**, 47, 5910-5912

5. J. C. Kistemaker, S. F. Pizzolato, T. van Leeuwen, T. C. Pijper, B. L. Feringa, *Chem. Eur. J.* **2016**, 22, 13478-13487

6. A. Ryabchun, F. Lancia, J. Chen, D. Morozov, B. L. Feringa, N. Katsonis, *Adv. Mater*. **2020**, 32, 2004420.

7. W. Danowski, T. van Leeuwen, S. Abdolahzadeh, D. Roke, W. R. Browne, S. J. Wezenberg, B. L. Feringa, *Nat. Nanotechnol*. **2019**, 14, 488-494.

8. M. Klok, M. Walko, E. M. Geertsema, N. Ruangsupapichat, J. C. Kistemaker, A. Meetsma, B. L. Feringa, *Eur. J. Chem*. **2008**, 14, 11183-11193.

9. a) G. Snatzke, *Circular Dichroism, Principles and Applications*, N. Berova, K. Nakanishi, R.W. Woody Eds., 2nd ed., Wiley-VCH, New York, 2000. b) N. Berova, L. di Bari, G. Pescitelli, *Chem. Soc. Rev.* **2007**, 36, 914-931.

10. S.C.J. Meskers, *ChemPhotoChem*, **2022**, 6(1), e202100154.

11. P. R. Gerber, *Zeitschrift für Naturforschung A*, **1980**, 35, 619-622

12. S. Leyre, E. Coutino-Gonzalez, J. Joos, J. Ryckaert, Y. Meuret, D. Poelman, P. Smet, G. Durinck, J. Hofkens, G. Deconinck, *Sci. Instrum.* **2014**, 85, 123115
